# Timing-dependent effects of salicylic acid treatment on phytohormonal changes, ROS regulation, and antioxidant defense in salinized barley (*Hordeum vulgare* L.)

**DOI:** 10.1038/s41598-020-70807-3

**Published:** 2020-08-17

**Authors:** Hülya Torun, Ondřej Novák, Jaromír Mikulík, Aleš Pěnčík, Miroslav Strnad, Faik Ahmet Ayaz

**Affiliations:** 1 Faculty of Agriculture and Natural Science, Düzce University, Düzce, Turkey; 2 Faculty of Science, Karadeniz Technical University, Trabzon, Turkey; 3 Laboratory of Growth Regulators, Faculty of Science, Palacký University and Institute of Experimental Botany, The Czech Academy of Sciences, Šlechtitelů 27, 78371 Olomouc, Czech Republic

**Keywords:** Plant physiology, Salt

## Abstract

Cross-talk between exogenous salicylic acid (SA) and endogenous phytohormone pathways affects the antioxidant defense system and its response to salt stress. The study presented here investigated the effects of SA treatment before and during salt stress on the levels of endogenous plant growth regulators in three barley cultivars with different salinity tolerances: *Hordeum vulgare* L. cvs. Akhisar (sensitive), Erginel (moderate), and Kalaycı (tolerant). The cultivars’ relative leaf water contents, growth parameters, proline contents, chlorophyll a/b ratios, and lipid peroxidation levels were measured, along with the activities of enzymes involved in detoxifying reactive oxygen species (ROS) including superoxide-dismutase, peroxidase, catalase, ascorbate-peroxidase, and glutathione-reductase. In addition, levels of several endogenous phytohormones (indole-3-acetic-acid, cytokinins, abscisic acid, jasmonic acid, and ethylene) were measured. Barley is known to be more salt tolerant than related plant species. Accordingly, none of the studied cultivars exhibited changes in membrane lipid peroxidation under salt stress. However, they responded differently to salt-stress with respect to their accumulation of phytohormones and antioxidant enzyme activity. The strongest and weakest increases in ABA and proline accumulation were observed in Kalaycı and Akhisar, respectively, suggesting that salt-stress was more effectively managed in Kalaycı. The effects of exogenous SA treatment depended on both the timing of the treatment and the cultivar to which it was applied. In general, however, where SA helped mitigate salt stress, it appeared to do so by increasing ROS scavenging capacity and antioxidant enzyme activity. SA treatment also induced changes in phytohormone levels, presumably as a consequence of SA-phytohormone salt-stress cross-talk.

## Introduction

Cereals are the main source of nutrients for all societies around the world. However, these plants are often grown under adverse environmental conditions. Barley (*Hordeum vulgare* L.) is an ancient grain that has become one of the most widely cultivated cereals^1^. Since the time of the Sumerians, it has been used as a foodstuff and for malting and brewing; more recently, it has also been used in biodiesel production. It can easily adapt to diverse environmental conditions, and is considered to be particularly salt tolerant for a member of the Triticeae^2^.

Salinity is one of the major abiotic stresses that limit plant growth, development, yield and food production^3^; it affects approximately 397 million ha of land worldwide^4^. Saline stress causes the formation of reactive oxygen species (ROS) such as the superoxide radical anion (O_2_.-), singlet oxygen (^1^O_2_), and hydrogen peroxide (H_2_O_2_)^5^, which trigger cascades of biochemical and physiological reactions that can lead to the induction of resistance and plant adaptation to environmental stress^6^. During the photosynthesis and respiration processes, plants constantly produce several ROS species in mitochondria, chloroplasts, and peroxisomes. However, overproduction of ROS adversely affects metabolic oxidative processes including the oxidation of organic molecules such as amino acids, lipids and DNA^7^. Plant cells have therefore developed various defense systems for scavenging and detoxifying ROS, including enzymatic antioxidant systems such as superoxide dismutase (SOD), peroxidase (POX), catalase (CAT), ascorbate peroxidase (APX), and glutathione reductase (GR), as well as non-enzymatic antioxidants such as glutathione, ascorbic acid, carotenoids, and tocopherols^8^.

Phytohormones, which occur in very low concentrations in plants, are key regulators of plant growth and development under stress conditions. In particular, they are known to regulate plants’ interactions with their environment^9^. Cytokinins (CKs), auxins, gibberellins (GA), and brassinosteroids (BRs) are known mainly as positive growth regulators while abscisic acid (ABA), jasmonic acid (JA) and ethylene are considered more as stress hormones^10^. To cope with salinity and increase stress resistance, plants may alter their ion metabolism, accumulate compatible solutes, activate antioxidant defense system, induce endogenous phytohormones, and alter their membrane structures^11^.

Plants have been exposed to growth regulators to clarify their stress tolerance mechanisms and the role of growth regulators in these mechanisms. To improve salt tolerance and mitigate the adverse effects of salt stress, several researchers have investigated the physiological, biochemical, and molecular responses of various plant species to treatment with exogenous phytohormones including indole-3-acetic acid (IAA), CK, GAs, ABA, JA, BR, and ethylene^12–16^. Salicylic acid (SA), which is considered to be a hormone-related substance^17^ is another endogenous signaling molecule that is important in regulating abiotic stress responses in plants^18^. More recent studies have examined the effects of treatment with exogenous SA on physiological and biochemical processes including antioxidant mechanisms, seed germination, and plant growth under the influence of salt stress^3,19,20^. SA was also found to alleviate the effects of salt stress on the growth and development of barley^21^. Crosstalk between plant hormones based on synergistic and antagonistic interactions plays an important role in abiotic stress responses^22^. The dynamics of endogenous phytohormones in plants under saline conditions have been studied previously^23,24^, but not in plants treated with exogenous growth regulators. Two studies have examined the mode of action of SA under saline conditions; one focusing on the accumulation of ABA, IAA, and selected CKs in wheat seedlings^25^, and one focusing on the accumulation of ABA and IAA in corn^26^.

Phytohormones also affect ROS formation and/or detoxification processes^24,27^. However, the mechanism of tolerance to salt-induced oxidative stress has yet not been fully described in terms of the dynamics of endogenous phytohormones. It is also unclear how the SA-regulated responses alter endogenous phytohormone crosstalk and how plants adapt to salinity via the antioxidant defense system under such conditions. Moreover, the specific knowledge on how SA signalling promotes salt tolerance and protects plants during salinity stress remain obscure^28^. Therefore, the aim of this study was to measure the time dependence of the changes in physiological parameters that can decide the salt stress tolerance in the presence and absence of SA treatment, and to investigate these effects on endogenous phytohormone levels. Future agriculture will require the identification and characterization of stress-tolerant cultivars of key crops. This in turn will require a detailed understanding of plant stress responses and the mechanisms by which plants adapt to salinity in terms of endogenous phytohormone regulation, ROS formation and detoxification. This work therefore examines the effects of salinity and exogenous SA treatment on the growth of three barley cultivars. To this end, growth parameters, relative water content (RWC), Chl *a/b* ratios, lipid peroxidation, and the activity of SOD, POX, CAT, APX, and GR were measured in barley seedlings grown under non-saline, moderately saline, and highly saline conditions, with and without exogenous SA. In addition, ROS generation and/or detoxification process in the seedlings were monitored and their levels of endogenous phytohormones such as IAA, CKs ABA, JA, and ethylene were determined.

## Results

### Growth parameters

**Figure 1:**
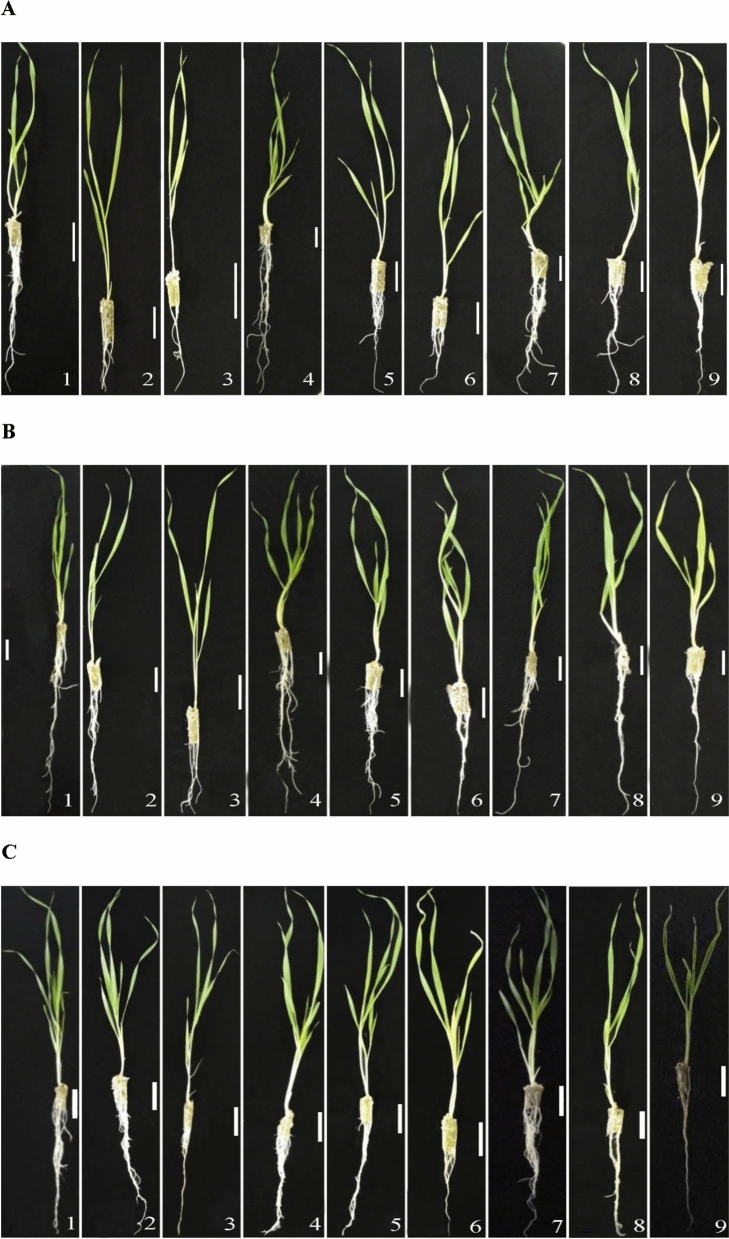
The effect of pre- and co-treatment with salicylic acid (SA) on the growth of barley cultivars under control and saline (150 and 300 mM NaCl) conditions. (**A**) *Hordeum vulgare* L. cv. Akhisar, (**B**) *Hordeum vulgare* L. cv. Erginel, (**C**) *Hordeum vulgare* L. cv. Kalaycı. Treatments: (1) Control, (2) 150 mM NaCl, (3) 300 mM NaCl, (4) 0.5 mM SA pre-treatment without NaCl stress, (5) 0.5 mM SA pre-treatment with 150 mM NaCl, (6) 0.5 mM SA pre-treatment with 300 mM NaCl, (7) 0.5 mM SA co-treatment without NaCl stress, (8) 0.5 mM SA co-treatment with 150 mM NaCl, (9) 0.5 mM SA co-treatment with 300 mM NaCl. (Scale bar, 5 cm).

**Figure 2:**
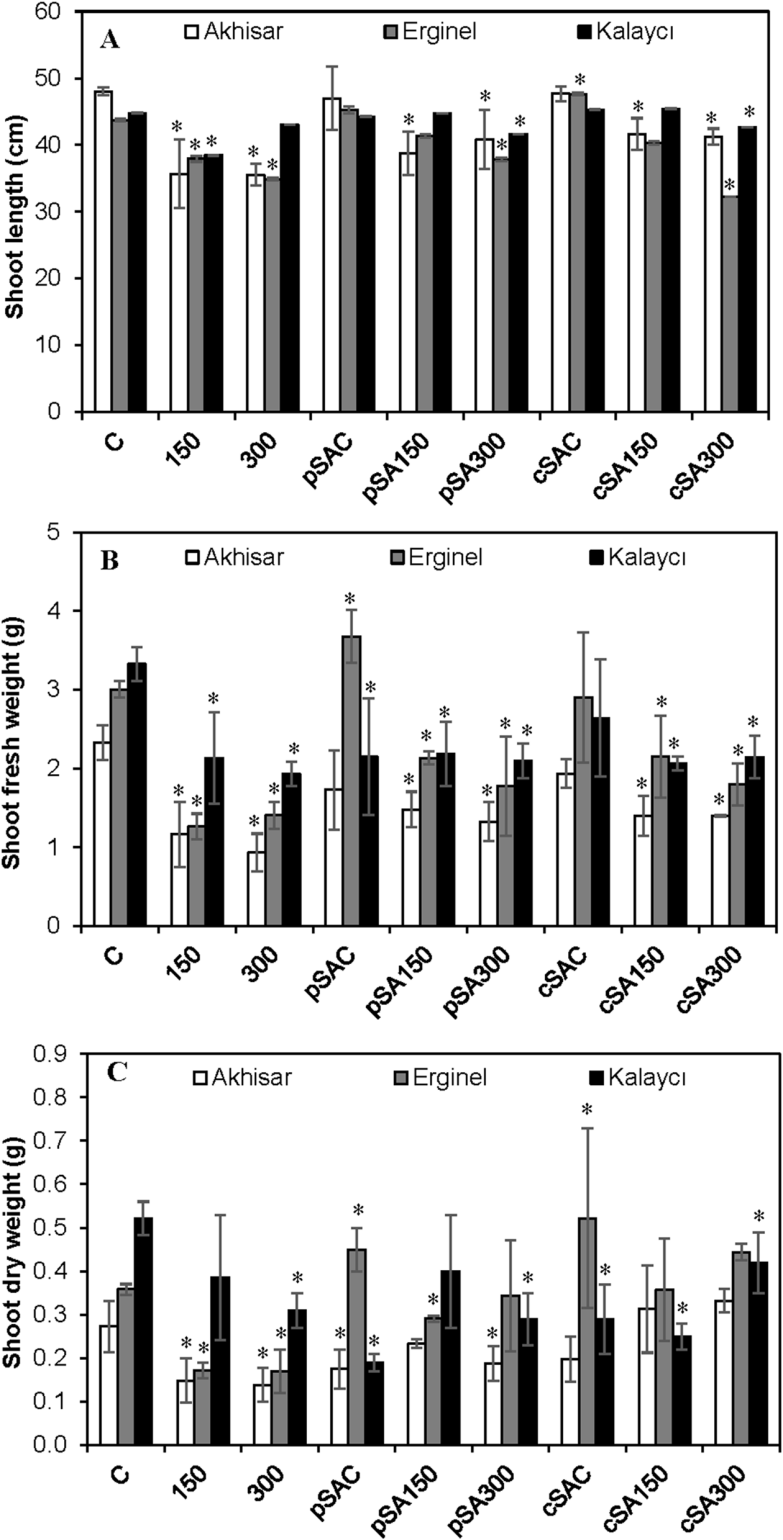
The effects of pre- and co-treatment with salicylic acid (SA) on the (**A**) shoot length (L; cm), (**B**) shoot fresh weight (FW; g) and (**C**) shoot dry weight (DW; g) in leaves of barley cultivars under control and saline (150 and 300 mM NaCl) conditions. Asterisks denote significant differences from controls (*P* < 0.05).

In this study we investigated the effects of SA treatment before and during salt stress on the different growth and physiological parameters in three barley cultivars with different salinity tolerances: Akhisar (sensitive), Erginel (moderate), and Kalaycı (tolerant). The barley seedlings were randomly divided into nine groups: C (untreated control in non-saline conditions); 150 (plants grown in 150 mM NaCl for 4 days); 300 (growth in 300 mM NaCl for 4 days); pSAC (pre-treatment with 0.5 mM SA for 24 h followed by growth without NaCl); pSA150 (pre-treatment with 0.5 mM SA for 24 h followed by growth in 150 mM NaCl); pSA300 (similar conditions but growth in 300 mM NaCl); cSAC (continuous treatment with 0.5 mM SA for 4 days while growing under non-saline conditions); cSA150 (continuous treatment with 0.5 mM SA for 4 days while growing in 150 mM NaCl) and cSA300 (similar conditions but growth in 300 mM NaCl). We measured first growth parameters including the shoot length (L) and fresh (FW) and dry weight (DW) of all three barley cultivars were adversely affected by salt stress (Figs. 1, 2 and Supplementary Table S1). All three cultivars exhibited reductions in FW and DW under salt stress. Relative to the non-saline control treatment (C), treatment with 150 mM NaCl caused slight reductions in L; treatment with 300 mM NaCl induced no significant reduction in L for Kalaycı but caused reductions of 26% and 20.3% in Akhisar and Erginel, respectively. Exposure to 300 mM NaCl reduced FW and DW accumulation in the Akhisar, Erginel and Kalaycı cultivars by 60.1–48.1%, 53.0–52.8% and 42.0–40.4%, respectively. Treatment with exogenous SA under non-saline conditions increased the L of Erginel but not that of Akhisar and Kalaycı, and also significantly reduced the FW and DW of the latter cultivars. It also significantly reduced the FW and DW of Akhisar and Kalaycı while increasing those of the Erginel plants.

Pretreatment with SA increased all growth parameters measured in this study for all three cultivars under saline conditions. For example, the shoot lengths of Akhisar and Erginel seedlings under the pSA150 and pSA300 treatments were 8.7–14.9% and 9.2–8.3% higher than those under the 150 and 300 treatments, respectively. Similarly, the shoot length of Kalaycı seedlings under the pSA150 treatment was 14.8% higher than that under the 150 treatment. SA pretreatment also increased the FW and DW of Akhisar and Erginel at both salt concentrations: the fresh weights of Akhisar and Erginel seedlings under the pSA150 and pSA300 treatments were 26.5–41.9% and 69.0–25.5% higher, respectively, than those under the 150 and 300 treatments without exogenous SA. The corresponding DW values were 53.3–35.7% and 70.6% higher, respectively, than those for controls without exogenous SA. SA pretreatment for 24 h also increased the FW of Kalaycı by 2.3% at 150 mM and 8.3% at 300 mM. However, these changes were not statistically significant.

Continuous SA treatment for 4 days (cSAC) had similar effects to pre-treatment with SA for 24 h. Akhisar and Kalaycı seedlings subjected to continuous SA treatment under non-saline conditions exhibited reductions in growth parameters. Conversely, Erginel seedlings exhibited increased growth under these conditions. Under saline conditions, continuous SA treatment generally increased growth compared to controls of the same salinity without exogenous SA. For example, the shoot lengths of Akhisar seedlings under the cSA150 and cSA300 treatments were 16.8 and 16.3% higher, respectively, than those for the corresponding controls. Similarly, Erginel and Kalaycı shoot lengths under the cSA150 treatment were 6.3 and 18.2% higher, respectively, than those for the same cultivars under 150 mM salt stress without exogenous SA. Like SA pretreatment, continuous SA treatment increased the FW and DW of all three cultivars under saline conditions, although the increase in DW was generally more pronounced than that in FW. The FW of Akhisar and Erginel increased by 19.7–6.1% and 70.6–27.7% under the cSA150 and cSA300 treatments, respectively, while their DW increased 2.1–2.4-fold and 2.1–2.6-fold. Kalaycı exhibited more modest increases; its FW and DW rose by 10.9% and 35.5%, respectively, under the cSA300 treatment.

To summarize, seedlings treated with SA under saline conditions (150 and 300 mM NaCl) exhibited the following changes in their growth parameters relative to controls not treated with SA: L increased except under the pSA150 and cSA300 treatments for Kalaycı and the cSA300 treatment for Erginel. Both FW and DW increased for all three cultivars, but the DW increase induced by SA pretreatment was less pronounced than that induced by continuous treatment. Strong DW increases were observed for Akhisar and Erginel; the increase observed for Kalaycı was less pronounced because this cultivar was less salt-sensitive to begin with. Each cultivar thus exhibited distinct responses to exogenous SA under salt stress, and these responses seemed to partially offset the adverse effects of salinity on the seedlings’ growth parameters. Of the three cultivars, Akhisar was probably the most responsive to the protective treatment.

### Leaf relative water content (RWC)

**Figure 3:**
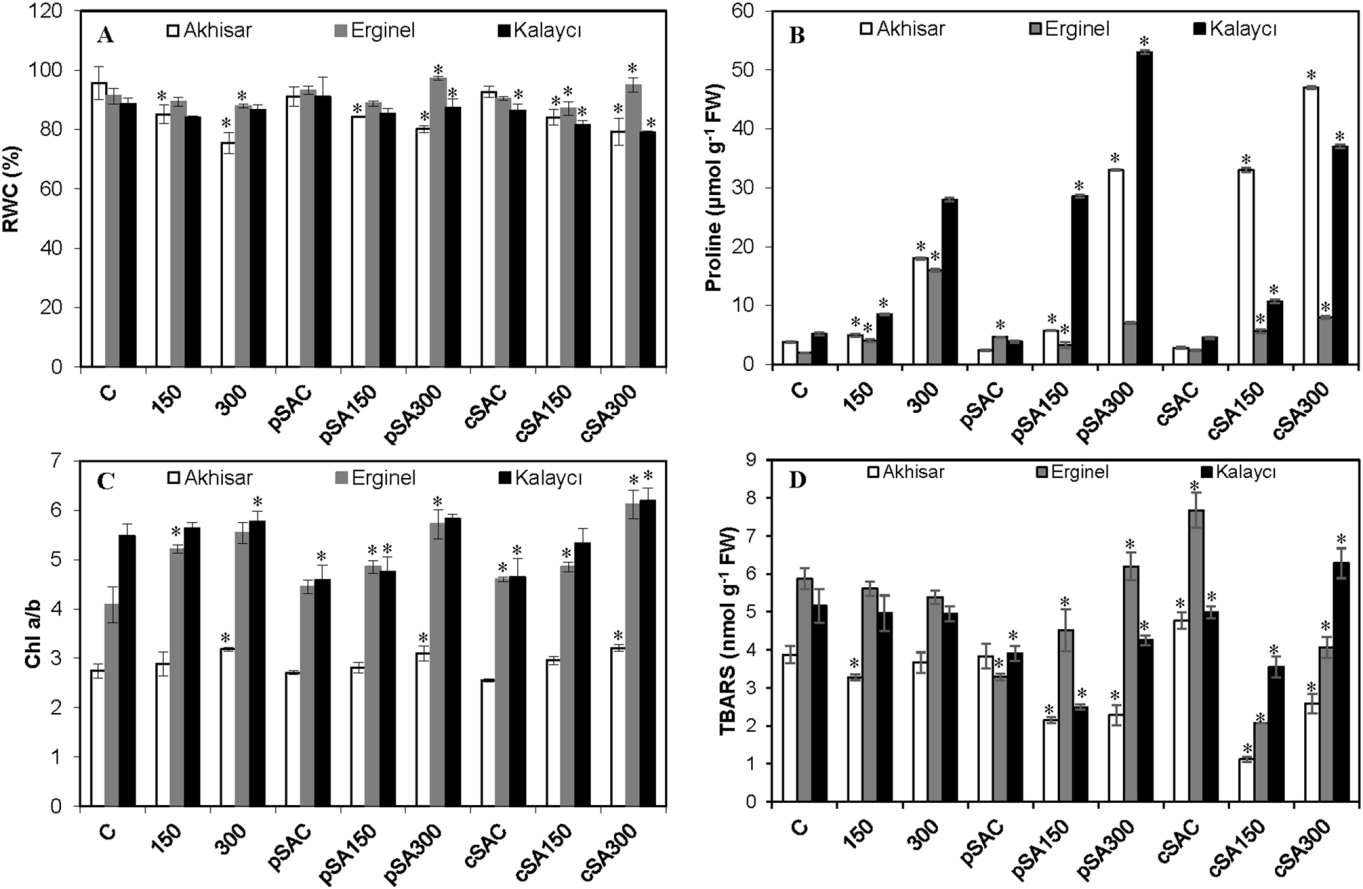
The effect of pre- and co-treatment with salicylic acid (SA) on the (**A**) relative water content (RWC), (**B**) proline content, (**C**) chlorophyll *a/b* ratio, and (**D**) TBARS level in leaves of barley cultivars under control and saline (150 and 300 mM NaCl) conditions. Asterisks denote significant differences from controls (*P* < 0.05).

The barley cultivars’ leaf RWC values were similar, ranging from 95.6% to 88.7%, and decreased with increasing salt stress (Fig. 3A). The most sensitive cultivar was Akhisar; its RWC values at NaCl concentrations of 150 mM and 300 mM were 10.9% and 21% lower, respectively, than those under non-saline conditions. The RWC of Erginel at 150 mM NaCl did not differ significantly (*P* < 0.05) from that of controls, but was 3.7% lower at 300 mM NaCl. SA pretreatment and simultaneous SA treatment had no significant effect on RWC. Both SA treatments increased the leaf RWC of Erginel at 300 mM NaCl (10.7 and 8.1%), but the pSA150 and cSA150 treatments had little effect. In Kalaycı, the RWC decreased under the cSA150 and cSA300 treatments, falling by 8.8% in the latter case. It thus appears that saline conditions reduce RWC in barley cultivars other than Erginel, and that this trend may be difficult to reverse by treatment with exogenous SA.

### Changes in proline content

Salt stress significantly (*P* < 0.05) increased the proline contents of all three barley cultivars (Fig. 3B). The 300 mM NaCl treatment increased the proline contents of Akhisar, Erginel and Kalaycı by factors of 3.8, 8.0 and 5.6 relative to control plants (C), respectively. However, neither of the exogenous SA treatments significantly affected the proline contents of any cultivar under non-saline conditions. Elevated salt concentrations increased the proline contents of all three cultivars independently of the SA regime. SA generally reduced proline accumulation in Erginel under saline conditions: its proline content under the pSA150 regime was around 25% lower than under the 150 regime, and its proline contents under the pSA300 and cSA300 regimes were 56.3–50% lower than under the 300 regime. The other two cultivars reacted quite differently: under the pSA150 and cSA150 regimes with NaCl concentrations of 150 mM, the proline contents of Akhisar seedlings were 20% and 660% higher, respectively, than under the SA-free 150 regime. The corresponding increases for the Kalaycı cultivar were 360% and 22.2%, respectively. At an NaCl concentration of 300 mM (i.e. under the pSA300 and cSA300 regimes), SA pre-treatment increased the proline content of Kalaycı by 89.3% relative to the SA-free 300 mM NaCl control, while continuous SA treatment increased the proline content of Akhisar by 310%.

### Chlorophyll *a/b* content

The Chl *a/b* ratio in control Kalaycı seedlings was considerably higher than that in Akhisar and Erginel seedlings (Fig. 3C). Similar ratios were maintained in all three cultivars under all tested conditions. The Chl *a/b* ratio increased by about 16.0% at 300 mM NaCl in Akhisar and by 27.5% and 35.7% in Erginel at 150 and 300 mM NaCl, respectively. However, there was barely any change in the Chl *a/b* ratio in Kalaycı at either salt concentration. Under non-saline conditions, pre- or simultaneous treatment with exogenous SA had no significant effect on the Chl *a/b* ratio in Akhisar. However, both SA treatments slightly increased this ratio in Erginel under non-saline conditions (by 8.9% and 12.6%, respectively) but reduced it significantly in Kalaycı (by 16.4% and 15.2%, respectively). Under saline conditions, SA treatment generally increased the Chl *a/b* ratio. The magnitude of these increases was comparable to that of the changes induced by varying the NaCl concentration. However, at a salt concentration of 150 mM, SA-NaCl co-treatment did not compensate for the negative effect of SA alone on the Chl *a/b* ratio in Kalaycı. The highest Chl *a/b* ratios were observed under the cSA300 regime, which restored the Chl *a/b* ratio of the Kalaycı cultivar to that seen in control (C) plants.

### TBARS content

The content of thiobarbituric acid-reactive substances (TBARS, nmol g^-1^ FW) in plant tissues is an indicator of the level of lipid peroxidation. Under control conditions, the TBARS contents of the three cultivars clearly differed. Salt treatment generally caused only non-significant reductions in the cultivars’ TBARS contents, although the TBARS content of Akhisar at 150 mM NaCl was 15.4% lower than in control plants (Fig. 3D). Saline conditions also caused a weak dose-dependent decrease in the TBARS content of Erginel. The two SA treatment regimens induced various changes in lipid peroxidation. Pre-treatment with exogenous SA under non-saline conditions significantly reduced the TBARS contents of Erginel and Kalaycı relative to controls (by 44.1 and 25.0%, respectively) but had no effect on that in Aksihar. Conversely, continuous SA treatment under non-saline conditions increased the TBARS contents of all cultivars other than Erginel. At an NaCl concentration of 150 mM, SA pre-treatment reduced lipid peroxidation relative to SA-free controls by 33.3%, 19.6%, and 50.0% in Akhisar, Erginel and Kalaycı, respectively. At an NaCl concentration of 300 mM, SA pretreatment increased lipid peroxidation by 14.8% in Erginel. However, the TBARS content of pSA300 Kalaycı plants was comparable to that in the corresponding control (300). Seedlings subjected to simultaneous SA + NaCl treatment generally exhibited significantly lower TBARS levels than SA-free controls (C, 150, 300) and cSAC plants. The only exception was Kalaycı, whose TBARS content under the cSA300 regime was significantly (28.6%) higher than that in the corresponding control.

### Antioxidant enzyme activities

**Figure 4:**
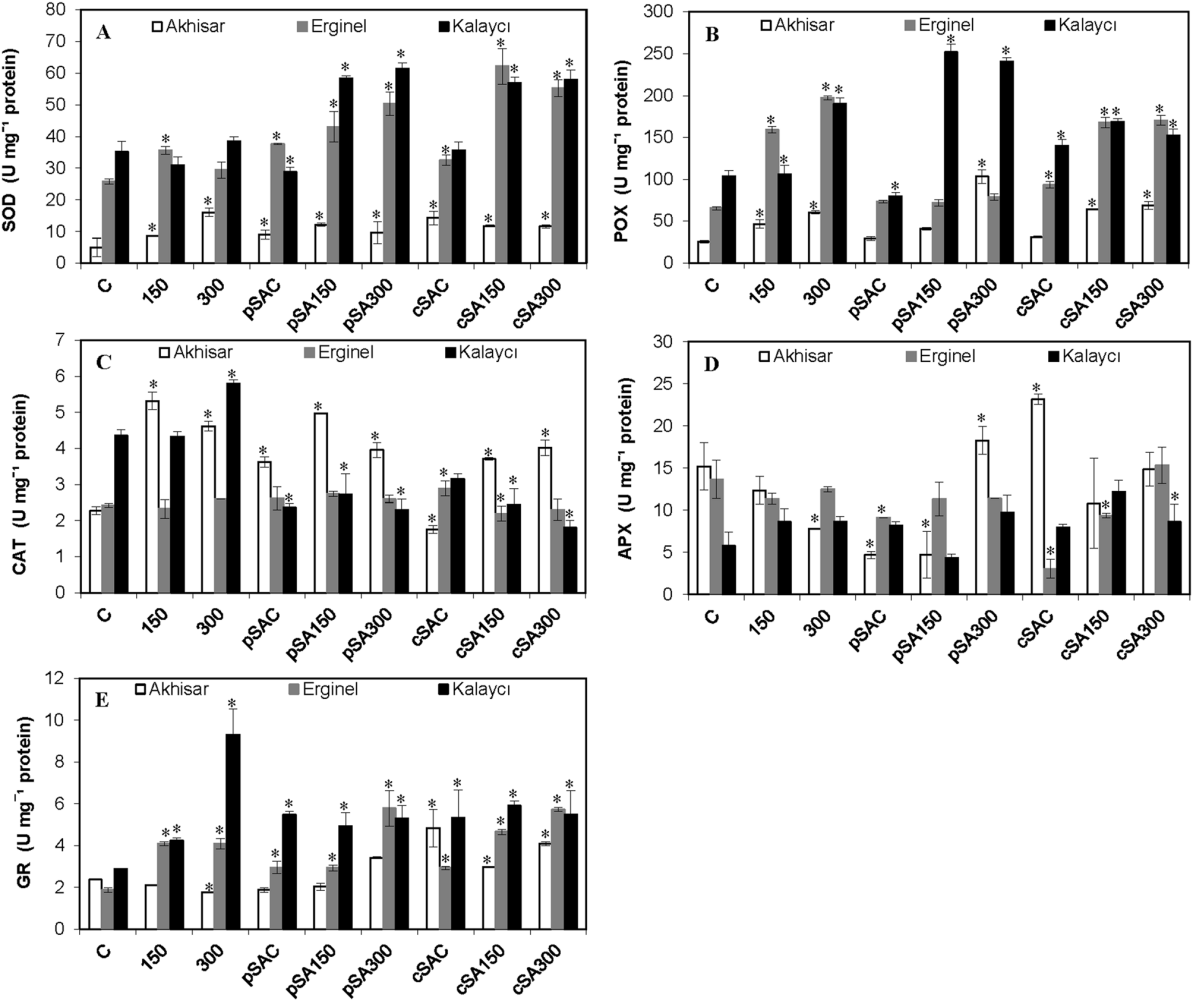
The effect of pre- and co-treatment with salicylic acid (SA) on (**A**) SOD, (**B**) POX, (**C**) CAT, (**D**) APX and (**E**) GR activities in leaves of barley cultivars under control and saline (150 and 300 mM NaCl) conditions. Asterisks denote significant differences from controls (*P* < 0.05).

The antioxidant enzyme activities of the three barley cultivars under control and experimental conditions are shown in Fig. 4. Under non-saline control conditions, superoxide dismutase (SOD) activity was significantly lower in Akhisar than in the two other cultivars (Fig. 4A). This trend persisted under all experimental conditions. Salinity had weak and variable effects on SOD activity in Erginel and Kalaycı but enhanced that in Akhisar leaves by 72.0% and 320% at NaCl concentrations of 150 and 300 mM, respectively. Under non-saline conditions, SA treatment increased SA activity in Akhisar and Erginel plants but reduced that in Kalaycı. However, exogenous SA treatment significantly increased SOD activity in all cultivars under saline conditions. The SOD activity of Akhisar under the different saline conditions with SA treatment varied significantly with no clear trends. At 150 mM NaCl, SA pre- or co-treatment increased SOD activity in Erginel relative to C plants by 21.1% and 88.7%, respectively, and increased that in Kalaycı by 74.4% and 84.1%, respectively.

As also observed for SOD activity, peroxidase (POX) activity under non-saline control conditions was highest in Kalaycı and lowest in Akhisar (Fig. 4B). In the absence of SA treatment, salt stress significantly increased POX activity—by 240%, 300%, and 83.4% in Erginel, Kalaycı, and Akhisar, respectively, at 300 mM NaCl. Under non-saline conditions, SA pre- or co-treatment had no significant effect on POX activity in Akhisar but caused significant increases (of 12.5% and 42.8%, respectively) in Erginel. In addition, SOX activity in Kalaycı under the cSA regime was 35.5% higher than in control (C) plants. Pre-treatment with exogenous SA strongly stimulated POX activity in Kalaycı under saline conditions but reduced that in Erginel by 54.8% at 150 mM NaCl and 60.1% at 300 mM NaCl. The effects of simultaneous SA treatment were more variable. In Akhisar, the pSA300 treatment increased POX activity by 70.3% relative to the corresponding SA-free control.

Under non-saline control conditions, catalase (CAT) activity was significantly higher in Kalaycı (91.3%) than in Erginel and Akhisar (Fig. 4C). Treatment with 150 and 300 mM NaCl had no effect on CAT activity in Erginel. However, this enzyme’s activity in Kalaycı increased by 31.8% at 300 mM NaCl while that in Akhisar was 230% higher than in controls at 150 mM NaCl and 200% higher at 300 mM NaCl. Independently of the salinity, co- or pre-treatment with exogenous SA had no effect on CAT activity in Erginel leaves but generally inhibited it in Kalaycı and Akhisar. However, under the pSAC regime, CAT activity in Akhisar was 56.5% higher than in the SA-free control.

Under non-saline control conditions, ascorbate peroxidase (APX) activity was highest in Akhisar and lowest in Kalaycı (Fig. 4D). Its activity gradually decreased as the NaCl concentration increased in Akhisar (by around 50% at 300 mM NaCl) and was also slightly reduced by salinity (dose-independently) in Erginel. However, its activity increased with salinity in Kalaycı. Pre- or co-treatment with SA generally inhibited this enzyme, independently of the NaCl concentration. However, the APX activity under the cSA300 regime was generally similar to that in the controls.

The three cultivars had similar glutathione reductase (GR) activity levels under non-saline control conditions. As shown in Fig. 4E, salinity slightly reduced GR activity relative to controls (by 25.4% at 300 mM NaCl) in Akhisar, induced strong (2.2-fold) increases at both 150 mM and 300 mM NaCl in Erginel, and caused strong dose-dependent increases in Kalaycı (1.5-fold at 150 mM NaCl and 3.2-fold at 300 mM). Both SA treatments stimulated GR activity, particularly the cSAC treatment. In addition, strong increases were observed at 300 mM NaCl in SA-treated Erginel and Akhisar plants. Interestingly, all the studied SA + NaCl treatments induced similar overall levels of GR activity.

### Phytohormone levels

**Figure 5:**
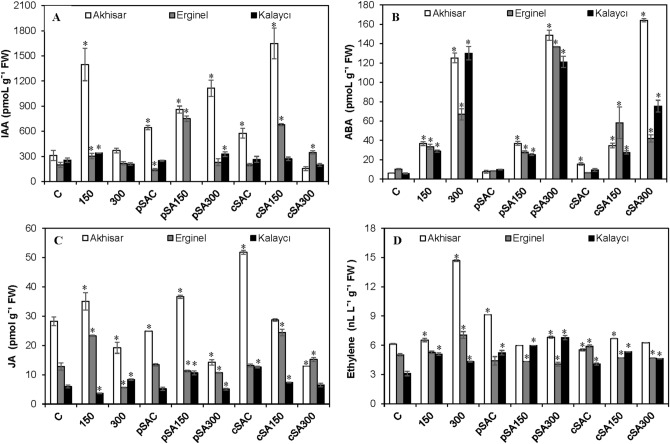
The effect of pre- and co-treatment with salicylic acid (SA) on (**A**) indole-3-acetic acid (IAA), (**B**) absisic acid (ABA), (**C**) jasmonic acid (JA) and (**D**) ethylene levels in leaves of barley cultivars under control and saline (150 and 300 mM NaCl) conditions. Absence of a column indicates the limit of detection. Asterisks denote significant differences from controls (*P* < 0.05).

**Figure 6:**
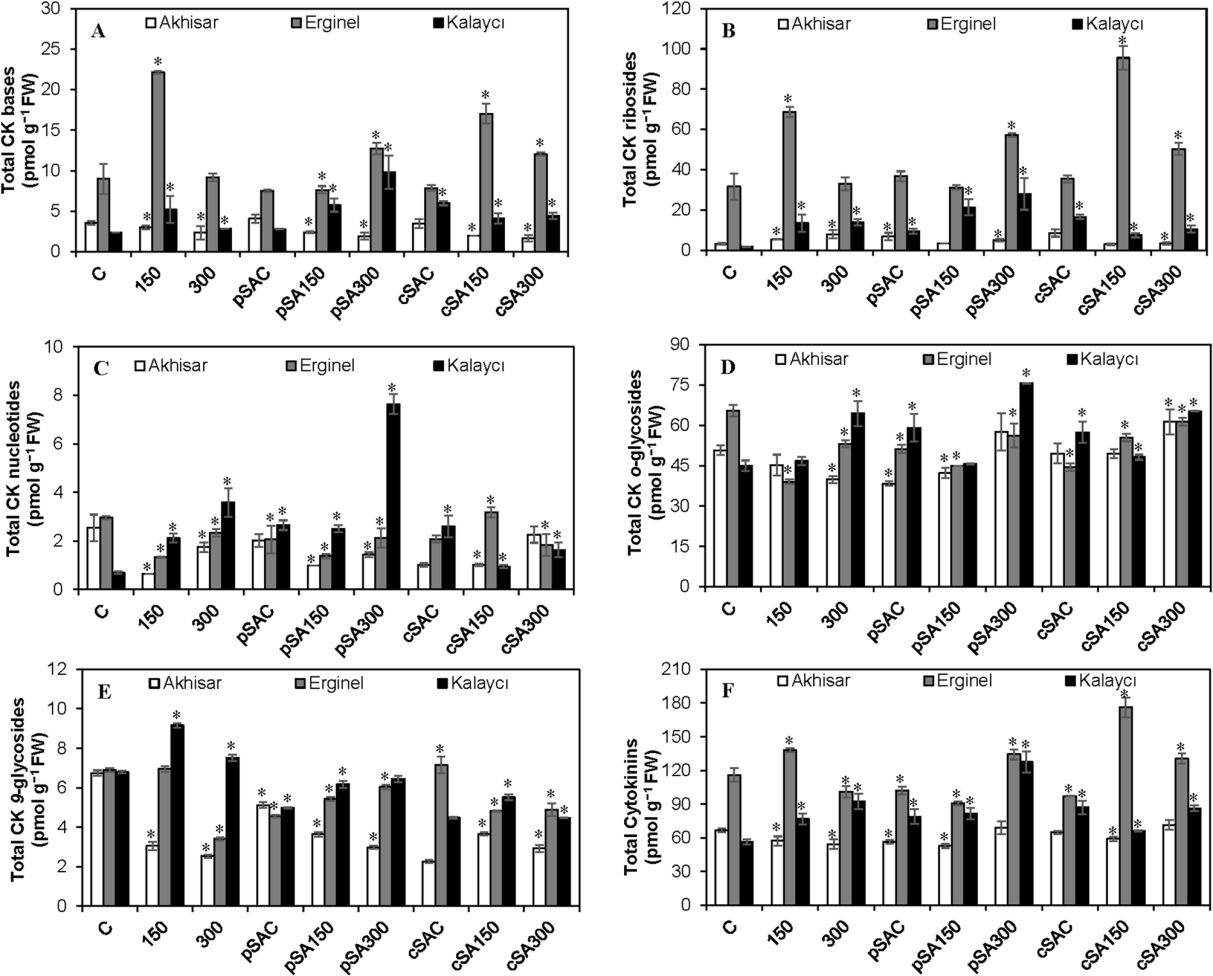
The effect of pre- and co-treatment with salicylic acid (SA) on endogenous cytokinin (CK) (**A**) bases, (**B**) ribosides, (**C**) nucleotides, (**D**) *o*-glycosides, (**E**) *9*-glycosides and (**F**) total cytokinins levels in leaves of barley cultivars under control and saline (150 and 300 mM NaCl) conditions. Asterisks denote significant differences from controls (*P* < 0.05).

The phytohormone responses of the barley cultivars to salt stress and pre- or co-treatment with SA were investigated by monitoring endogenous hormone levels in tissue samples using UHPLC-MS/MS (see Figs. 5 and 6).

Under non-saline control conditions, Akhisar had the highest indole-3-acetic acid (IAA) level but all three cultivars had similar IAA levels, which ranged from 199.7 to 311.4 pmol g^-1^ FW (Fig. 5A). The 150 mM NaCl treatment significantly increased IAA levels, particularly in Akhisar (which exhibited a 450% increase). However, the IAA levels in plants treated with 300 mM NaCl were very similar to those in control plants. SA pre- or co-treatment (pSAC or cSAC) under non-saline conditions increased IAA levels in Akhisar. However, the IAA level in Erginel fell slightly under the pSAC regime, while that in Kalaycı increased slightly under the cSAC regime. Under saline conditions (150 mM NaCl), pSAC treatment strongly increased IAA levels in Akhisar and Erginel (by 33.5% and 530%, respectively) relative to pSAC under non-saline conditions. However, only Erginel exhibited a significant increase relative to the level observed at 150 mM NaCl without exogenous SA. Conversely, the IAA content of Kalaycı under the pSA150 regime was below the detection limit. Relative to the 150 mM NaCl control, SA co-treatment at 150 mM NaCl increased IAA levels in Akhisar and Erginel (by 18.0% and 220%, respectively), but reduced those in Kalaycı by 20.2%. These IAA levels were all higher than those induced by SA co-treatment under non-saline conditions (cSAC). At 300 mM NaCl, no SA treatment had any significant effect on IAA levels with the exception of the pSA300 (which increased the IAA level of Aksihar leaves threefold) and cSA300 (which increased the IAA levels of Erginel leaves by 60.1% compared to 300 mM NaCl alone).

Abscisic acid (ABA) is a major stress-related phytohormone. Its concentration was lowest in Kalaycı leaves and highest in Erginel (Fig. 5B). Endogenous ABA concentrations ranged from 5.9. to 10.3 pmol g^-1^ FW and increased in a dose-dependent manner in response to salt stress in all three cultivars. However, the magnitude of the increase differed between cultivars: at 150 mM NaCl, ABA levels increased 5.9-, 3.3- and 4.9-fold in Akhisar, Erginel and Kalaycı, respectively, while 20.1-, 6.5- and 22.0-fold increases were observed at 300 mM NaCl. SA treatment did not significantly affect ABA levels in most cases, although the cSA150 treatment increased the ABA level in Erginel by 73.9% relative to the corresponding saline control. Exogenous SA treatment had stronger effects on ABA levels in plants under strongly saline conditions (300 mM NaCl), and both the magnitude and nature of these effects depended on the route of SA exposure and the cultivar. Pre- and co-treatment with SA increased ABA levels in Akhisar (by 18.8% and 31.1%, respectively) but reduced those in Kalaycı (by 6.9% and 42%, respectively). ABA levels in Erginel leaves increased by 81% (relative to salt-treated controls) under the pSA300 regime but were reduced by 37.3% under the cSA300 regime.

As shown in Fig. 5C, there were large differences in the jasmonic acid (JA) contents of the barley cultivars’ leaves. Under non-saline control conditions, the JA concentration in Akhisar (28.3 pmol g^-1^ FW) was around twice that in Erginel, which in turn was around twice that in Kalaycı (Fig. 4C). Under moderately saline conditions (150 mM NaCl), the JA levels in Akhisar and Erginel increased by 23.9 and 81.9%, respectively, while that in Kalaycı fell. The opposite occurred at 300 mM NaCl: JA levels in Akhisar and Erginel were lower (by 32.1% and 56.2%, respectively) than under non-saline control conditions, while those in Kalaycı were 38.8% higher. The JA levels under pSAC conditions were similar to those in the non-saline controls (C), while those under cSAC conditions were 83.3%, 270%, and 210% higher than in C for the Akhisar, Erginel, and Kalaycı cultivars, respectively. Under the pSA150 treatment, the JA contents of the Akhisar and Kalaycı cultivars were higher (by 18.1% and 290%, respectively) than under the 150 treatment, while that of Erginel was 51.8% lower. JA levels in Akhisar and Kalaycı were reduced significantly by SA pre-treatment at 300 mM NaCl while those in Erginel increased by 90%. Salinity also dose-dependently reduced JA levels in Akhisar and Kalaycı seedlings subjected to SA co-treatment; the JA contents of these cultivars under the cSA300 conditions were 18.1% and 22.5% lower, respectively, than under the cSA150 conditions. In Erginel, SA co-treatment at 300 mM NaCl increased JA levels by a factor of 2.7 relative to the 300 mM treatment without exogenous SA, but JA levels in seedlings co-treated with SA at 150 mM NaCl did not differ significantly from those observed at the same NaCl concentration without exogenous SA.

As with JA, ethylene levels (measured in nl L^−1^ g^-1^ FW) under non-saline control conditions were highest Akhisar and lowest in Kalaycı (Fig. 5D). However, the differences between the cultivars were less pronounced than for JA. The measured ethylene levels increased with salinity; the ethylene levels in Akhisar at 300 mM NaCl were 2.4-fold higher than under the control treatment, while Kalaycı and Erginel exhibited more modest increases of 38.6% and 40.6%, respectively. The pSAC and cSAC treatments caused minor increases in ethylene levels; this effect was most pronounced in Akhisar and Kalaycı under pSAC conditions. Ethylene levels under the pSA150 and cSA150 treatments differed only slightly from those observed at 150 mM NaCl without added SA. The ethylene contents of seedlings treated with SA at an NaCl concentration of 300 mM (pSA300 and cSA300) were generally lower than those of seedlings grown at the same salt concentration without exogenous SA; the greatest reduction (57.4%) was observed for the Akhisar cultivar. However, the ethylene content of the Kalaycı cultivar under the pSA300 treatment was 57.5% higher than that under the SA-free 300 mM control treatment.

The cytokinin (CK) metabolites determined in this study were the free bases (active forms), ribosides (transport forms), nucleotides (biosynthetic precursors), and glucosides (storage forms) of *trans*-zeatin (*t*Z), *cis*-zeatin (*c*Z) and isopentenyladenine (iP). Over 30 different metabolites were measured in leaves of barley cultivars, yielding the results shown in Fig. 6 and Supplementary Table S2. The cultivars differed in their overall cytokinin contents and in the relative abundance of individual forms. Under the control (C) treatment, the Erginel cultivar had the highest total CK content (116.0 ± 6.1 pmol g^−1^ FW), followed by the Akhisar and then Kalaycı cultivars. Erginel was unique in that it had unusually high levels of ribosides (31.68 ± 6.46 pmol g^−1^ FW) and free bases (8.99 ± 1.87 pmol g^−1^ FW). The distributions of individual forms were similar in all three cultivars, with *O*-glucosides being most abundant, followed by 9-glucosides, free bases, ribosides, and nucleotides. In all three cultivars, *cis*-zeatin-type cytokinins predominated, followed by *trans*-zeatin (*t*Z) and isopentenyladenine (iP) types (Supplementary Tables S3–S5). The salt treatments induced significant dose-dependent increases (of 35.7% and 64.3% at 150 and 300 mM NaCl) in the total CK levels in Kalaycı. Conversely, the total CK concentrations in Akhisar seedlings fell by 14.3% and 18.6% at 150 and 300 mM NaCl, respectively, while those in Erginel seedlings increased by 19.1% relative to the control at 150 mM NaCl but fell to 12.9% below the control level at 300 mM NaCl. High salt concentrations increased CK biosynthesis in Kalaycı, as indicated by a threefold increase in the abundance of the nucleotide form at 150 mM NaCl and a 5.1-fold increase at 300 mM NaCl. Ribosides appeared to be the CK form most sensitive to salinity because their abundance increased with the salt concentration in all cultivars. The Kalaycı cultivar exhibited a particularly strong cytokinin response to salinity: its CK riboside content increased 7.8- and 8.1-fold (relative to the non-saline control) at NaCl concentrations of 150 mM and 300 mM, respectively. The increase in riboside levels was generally accompanied by reductions in the abundance of endogenous *O*-glucosides and also 9-glucosides in some cases. However, the Kalaycı cultivar did not follow this pattern. Interestingly, the total active CK (free base) content of the Kalaycı and Erginel cultivars increased 2.2- and 2.5-fold at 150 mM NaCl but decreased by 15.4% in Akhisar at the same salt concentration. The cytokinin types with the strongest salt responses were the *c*Z and iP-types in Kalaycı (whose abundance increased by 67.2% and 500% at 300 mM NaCl, respectively; see Supplementary Table S5). The total levels of *c*Z and iP also increased in Erginel at 150 mM NaCl. However, salinity had no significant effect on the total *t*Z, *c*Z and iP levels of Erginel and Akhisar (Supplementary Tables S3 and S4).

Under non-saline conditions, the pSAC and cSAC treatments reduced the total CK contents of Akhisar and Erginel but increased that of Kalaycı by 39.5 and 54%, respectively. The only CK forms whose levels fell in Kalaycı upon treatment with exogenous SA were CK 9-glucosides. Stronger reductions were observed in the levels of CK 9-glucosides in pSAC and cSAC-treated Kalaycı and Ashisar seedlings, and in the levels of *O*-glucosides in Erginel. Treatment with exogenous SA under saline conditions generally increased overall CK levels, particularly at 300 mM NaCl. However, a slight reduction in CK levels was observed in Akhisar and Erginel under the pSA150 regime. SA co-treatment under saline conditions increased overall CK levels in all three cultivars; this was primarily due to increases in the abundance of *c*Z-types, but increases in the levels of *t*Z and iP types were also observed in Erginel and Kalaycı. The main cytokinin forms responsible for these increases were free bases and ribosides, as well as *O*- and 9-glucosides in some cases. Co-treatment with SA under saline conditions induced different cultivar-dependent cytokinin production patterns to those induced by SA pre-treatment. Interestingly, in the Kalaycı cultivar (which had a strong cytokinin response to all treatments), total CK levels under the cSA150 and cSA300 regimes were 13.7% and 6.7% lower, respectively, than those observed at the same NaCl concentrations without exogenous SA, and were also lower than those observed under non-saline conditions with SA co-treatment. Conversely, in Erginel leaves, the pSA150 and pSA300 treatments increased CK levels by 27.3% and 29.1%, respectively, relative to those observed under the corresponding SA-free saline conditions. Similar outcomes were observed for Akhisar leaves. These outcomes were mainly due to the presence of relatively large quantities of ribosides and *O*-glucosides in Akhisar and Erginel, while Kalaycı had relatively low levels of ribosides and 9-glucosides. It is also interesting that SA treatment under saline conditions reduced levels of *t*Z- and iP-type CKs below those seen in any control or saline treatment without exogenous SA. However, the production of *c*Z-type CKs was stimulated under all saline SA treatments other than the cSA150 treatment in the case of Kalaycı cultivar. These outcomes were mainly due to changes in the abundance of ribosides and *O*-glucosides.

## Discussion

Plants regulate their growth and development and adapt to environmental conditions via signal transduction pathways that integrate ROS and phytohormonal signaling networks^29^. This work explores the relationship between stress and phytohormone regulation in three barley cultivars after exposure to salt stress, and the responses of stress markers and phytohormones to exogenous salicylic acid (SA) under these conditions. One of the first signs of plant stress is reduced growth. Previous studies showed that salt stress reduces shoot length and fresh and dry weight in sesame^30^, alfalfa^31^ and tomato^32^. Salinity also changed the growth parameters of the barley cultivars examined here. Our results showed that Kalaycı was more tolerant of salinity than Akhisar and Erginel because salt stress had the smallest effect on its length and the fresh and dry weights of its shoots. This is consistent with the findings of Seckin et al.^2^, who reported that salt-tolerant cultivars retain more biomass under salt stress than do less tolerant cultivars. Our results are also consistent with previous studies on the growth of salt-sensitive cultivars^24,30,33^. Earlier investigations indicated that exogenous SA treatment can extend the survival of barley cultivars and maintain growth under salt stress. This response was demonstrated in wheat pre-treated with SA^19,21^, Arabidopsis^20^, and soybean^34^. None of these earlier studies investigated the effect of the timing of SA application or considered potential protective responses and their relationship to growth under salt stress. However, one group examined the time dependence of the effects of SA on the K^+^ and H^+^ transport systems^20^. Their results indicated that pre-treatment with SA for one hour reduced K^+^ and H^+^ influx under salt stress to a greater degree than simultaneous application of SA and salt stress. Our results are similar in that we observed growth induction due to SA treatment, although it was strongly cultivar-dependent. According to our results, growth parameters were most influenced by salinity for the cultivar Akhisar and least affected for the cultivar Kalaycı. Kalaycı thus appeared to be the most salt resistant cultivar. Furthermore, because Kalaycı exhibited comparatively low DW losses under saline conditions without exogenous SA, the protective effect of SA was less pronounced in this cultivar. The protective effect of pre-treatment with SA was generally weaker than that of continuous treatment. It is also important to note that SA strongly inhibited the growth of the Kalaycı cultivar, leading to reductions in all its growth parameters. In the current study, SA treatment might counterbalance the adverse effects of salt stress in all three barley cultivars in terms of growth parameters. Moreover, Akhisar, previously reported as a salt sensitive cultivar^35^, was probably the most responsive to SA effects.

In general, plants’ water contents reflect their health. The effect of salt stress on RWC was found to be much weaker in Kalaycı and Erginel (which retained as much as 85% of their leaf water when grown in 300 mM NaCl) than in Akhisar. The SA treatments caused no significant change in RWC. Previous studies also found that treatment with exogenous SA counteracted adverse salt-induced effects on RWC^20,21^. However, our results indicate that this outcome is highly cultivar-dependent. For example, the RWC of Akhisar seedlings was strongly dependent on the salt concentration, independently of SA treatment. Treatment with SA at an NaCl concentration of 300 mM increased the leaf RWC of Erginel seedlings, whereas that of Kalaycı leaves was reduced by SA co-treatment, particularly under the cSA300 conditions. The RWC of two of the barley cultivars thus fell as the NaCl concentration increased, and this trend could not always be reversed by treatment with SA.

The Chl *a/b* ratio is an indicator of stress damage in plants. In this work, the Chl *a/b* ratio varied between cultivars and was not affected by salt stress in the Kalaycı cultivar. These findings are consistent with an earlier study on maize in which salt stress did not alter photosynthetic pigment levels^36^. In addition, Islam et al.^37^ reported that barley is more salt-tolerant than other Triticeae members. These findings indicate that Kalaycı effectively protects its leaf photochemistry against salt stress. As with RWC, SA treatment increased Chl *a/b* ratio in certain cultivars under certain conditions. Notably, the Chl *a/b* ratios under the cSA300 regime were comparable to those in control plants. SA treatment administered at the same time as salt exposure thus increased the Chl *a/b* ratio, especially in Erginel. Chlorophyll concentrations have previously been reported to exhibit genotype-dependent responses to SA treatment^38^.

Plants achieve osmotic regulation by controlling the accumulation of water-soluble organic metabolites known as osmolytes. Proline is a particularly important osmolyte that accumulates under salt stress. Under non-saline conditions, SA treatment had no significant effect on proline levels in the studied cultivars. The Akhisar and Kalaycı cultivars increased their accumulation of proline under saline conditions but the Erginel cultivar did not. The observed accumulation of proline under saline conditions is consistent with observations of other plants such as wheat^21^ and sesame^30^. Treatment with exogenous SA significantly affected proline accumulation under saline conditions, and its effects depended on the timing of the treatment and cultivar (higher proline accumulation in the Kalaycı and Akhisar cultivars than in the Erginel cultivar). Previous studies also attributed the protective effect of SA to mitigation of the detrimental effects of salt stress resulting from proline accumulation^21,25^.

Recent publications suggest that increasing salinity stress results in increased lipid peroxidation^2,35,36^. However, in our study, salt treatment caused no significant changes or only slight reductions in the TBARS (thiobarbituric acid-reactive substances) contents of the three barley cultivars. This could have been due to the protective action of antioxidant defense systems. More salt-tolerant Kalaycı cultivar exhibited the strongest accumulation of proline and the lowest level of lipid peroxidation of the three cultivars included in the study. Cultivars in which the leaves maintain stable, salt-independent levels of lipid peroxidation are likely to be better protected against oxidative damage due to salt stress, and are thus more salt-resistant. In addition to improving leaf RWC levels and proline contents, pre-treatment with exogenous SA significantly reduced lipid peroxidation, indicating that SA delayed leaf senescence and suppressed oxidative damage in barley cultivars. However, the TBARS levels of the studied cultivars under simultaneous SA + NaCl treatment were significantly lower than under control conditions. The results obtained thus indicate that treating salt-stressed barley with exogenous SA affects vegetative growth parameters, RWC, the chlorophyll a/b ratio, and the contents of proline and TBARS, and that these effects are species-, cultivar-, and growth stage-dependent.

Salt stress increases cellular ROS production^7^. ROS are cellular indicators of stress and, as secondary messengers, are involved in the stress response and signal transduction pathways. In addition to their signaling role, ROS can damage cellular components. To manage this threat, plant antioxidant defense systems incorporate ROS-scavenging enzymes such as superoxide dismutase (SOD), peroxidase (POX), catalase (CAT), ascorbate peroxidase (APX) and glutathione reductase (GR). High levels of antioxidant enzyme activity protect plant cells and tissues against stress-induced oxidative damage. Previous studies have shown that ROS act as signals that activate salt stress response and defense pathways in plants, and that high levels of ROS production may be indicative of oxidative damage^8^. Under the control conditions used in this work, the activities of SOD, POX, CAT, and GR were higher in the Kalaycı cultivar than in the Erginel and Akhisar cultivars, while the opposite was true for APX activity. SOD catalyzes the conversion of the superoxide radical anion, O_2_^-^, into the more stable H_2_O_2._ Increasing salt stress enhanced SOD activity in the leaves of the Akhisar and Erginel cultivars but had no consistent effect on that in Kalaycı seedlings. Increases in SOD activity are associated with increases in O_2_^-^ production^39^, suggesting that salt stress increased O_2_^-^ production in the Akhisar and Erginel cultivars but not in the Kalaycı cultivar. This again indicates that Kalaycı is more salt-tolerant than the other two cultivars. In keeping with these results, Sekmen et al.^33^ and Torre-González et al.^24^ reported high basal SOD activity in salt-tolerant *Plantago maritima* and tomato (Grand Brix) cultivars. Whereas the SOD activity of the Kalaycı cultivar was largely independent of salt stress, its CAT and GR activities increased upon NaCl exposure. CAT activity also increased with salt stress in Akhisar, while GR activity increased under salt stress in Erginel. POX and CAT are the major H_2_O_2_ scavenging enzymes in plants, but CAT is only present in peroxisomes^7^. Under salt stress, POX activity declined in Kalaycı but increased in Akhisar and Erginel leaves. APX regulates H_2_O_2_ levels in the cytosol and chloroplasts. Like CAT, it uses ascorbate as the electron donor and catalyzes the degradation of H_2_O_2_ generated by SOD activity^40^. It also catalyzes the ascorbate–glutathione cycle together with GR, using glutathione as a substrate. Under salt stress, APX activity declined in Akhisar and Erginel but increased in Kalaycı. In keeping with these results, Mittler^7^ reported that plants with suppressed APX activity exhibit enhanced compensatory SOD, CAT and GR activity, while plants with CAT suppression exhibit enhanced APX and glutathione peroxidase (GPX) activity. Saline conditions slightly reduced or did not alter CAT and APX activity in Erginel but increased APX activity in Kalaycı. These data illustrate the importance of H_2_O_2_ scavengers other than APX, as indicated by the increased SOD, POX and GR activity in Erginel and the increased CAT and GR activity in Kalaycı leaves.

SA had genotype-specific and time-dependent effects on the regulation of the antioxidant defense system. Pre- and co-treatment with SA both altered this system’s effectiveness in barley but their effects were less pronounced in Akhisar than in Erginel and Kalaycı, both of which exhibited SA responses that were highly dependent on the NaCl concentration. More effective ROS scavenging was observed in Kalaycı pre-treated with SA and Erginel co-treated with SA. Exogenous SA treatment also increased the activity of APX and GR under saline conditions in Akhisar, and SA co-treatment increased SOD, APX, and GR activity under saline conditions in Erginel leaves. Similar results were obtained in studies on heat-treated *Agrostis stolonifera* by Larkindale and Huang^41^ and on tobacco by Chen et al.^42^ and Klessig et al.^43^, all of which showed that SA treatment inhibited CAT activity. However, with the exception of CAT, both SA treatments increased the activity of H_2_O_2_-scavenging enzymes relative to that in plants not treated with both SA and salt. These results are consistent with earlier studies showing that treatment with exogenous SA enhanced SOD, POX, APX, and GR activity under salt stress^44^. In contrast to our findings, Ma et al.^44^ observed increased CAT activity following SA treatment in *Dianthus superbus* under saline conditions.

We also examined the effect of the timing of SA treatment on phytohormone regulation and ROS signaling in barley because the effects of SA on phytohormone profiles under salt stress are poorly documented. However, ROS signaling is known to be tightly integrated with hormonal signaling networks^29^. Auxins play a key role in regulating aspects of plant growth and development including cell elongation, vascular tissue development, and apical dominance^45^. They also induce programmed and cell-specific ROS generation and regulate antioxidant levels^29^. IAA is the most common naturally occurring auxin. Barley seedlings exposed to 150 mM NaCl exhibited higher IAA levels than controls grown under non-saline conditions. However, IAA levels in seedlings exposed to 300 mM were very similar to those in control plants. The increase in IAA levels at 150 mM NaCl coincided with an increase in the Chl*a/b* ratio and reduced TBARS levels, neither of which were observed at the higher salt concentration. This is consistent with an earlier study on *Avena* coleoptiles^46^ treated with exogenous IAA, which caused a rapid decrease in lipid peroxidation. A separate study found that salinity reduced IAA levels in maize^25^, but another study on the same plant found the opposite^26^. In keeping with our results, salt stress reportedly increased IAA levels in tomatoes^24^. The inconsistencies in these findings could be due to differences in stress duration, NaCl concentration, or the properties of the studied species/cultivars. SA treatment under non-saline conditions increased IAA levels in Akhisar while those in Erginel decreased slightly under the pSAC conditions and those in Kalaycı increased slightly under the cSAC regime. Both Erginel and Akhisar exhibited also elevated IAA levels as a result of SA co-treatment under salt stress. Shakirova et al.^25^ found that SA treatment before sowing prevented SA-induced reductions in the IAA levels of wheat grown under saline conditions. Increasing IAA levels could reduce osmotic stress.

ABA has been extensively studied and is one of the most important plant growth regulators. It is considered to be a stress hormone because it is a key internal signal that enables plants to survive adverse environmental conditions^47^. The ABA concentration increased in all genotypes under saline conditions, particularly at the higher salt concentration (300 mM NaCl). The ABA levels in Kalaycı and Akhisar were much higher than those in Erginel. This may reflect the differences in salt tolerance between the cultivars. The high accumulation of ABA in Kalaycı could also help to reduce its rate of water loss. Similar results were reported by Amjad et al.^48^ and Torre-González et al.^24^, who found that salt-tolerant plants had significantly higher concentrations of ABA under saline conditions than less tolerant varieties. This response also suggests that there may be an interaction between proline and ABA accumulation. SA treatment did not significantly alter ABA levels under non-saline conditions or at an NaCl concentration of 150 mM. Both pre- and co-treatment with SA increased ABA levels in Akhisar but decreased them Kalaycı. In the case of Erginel, SA pre-treatment at 300 mM NaCl increased ABA levels by approximately 81% whereas SA co-treatment reduced them by 37.3%. The ABA/IAA ratio is an important indicator of salinity tolerance that typically increases under saline conditions in maize but was reduced by SA treatment in salt-stressed plants^26^. The highest ABA/IAA ratio was observed in Kalaycı, for which the ratio observed at 300 mM NaCl was 31 times higher than that under non-saline control conditions. SA pre-treatment also reduced the ABA/IAA ratio in all cases whereas cSA treatment reduced the ABA/IAA ratio in Erginel leaves but increased it in Kalaycı and Akhisar. The changes in ABA levels induced in Erginel leaves by exogenous SA treatment were comparable to those observed for growth-stimulating hormones such as IAA and CKs. Whereas levels of IAA and JA decreased at 300 mM NaCl, those of CKs and ABA increased under both the pSA300 and cSA300 conditions. These findings suggest that SA treatment can induce both antagonistic and agonistic interactions between different growth regulators. However, Pospíšilová^49^ reported that ABA can be regarded as a CK antagonist. In addition, antagonistic interactions between ABA and JA were observed in our experiments under highly saline conditions. Similar results have been observed in rice roots under salt stress^12^.

CKs can have both positive and negative effects on stress tolerance, depending on the plant species and the duration and intensity of the stress^50^. Salt induced a significant dose-dependent increase in total CK levels in Kalaycı but reduced CK levels in Akhisar. In Erginel, the content of total CKs increased at 150 mM NaCl but then fell below the control level at 300 mM NaCl. The cytokinin types with the strongest salt responses in Kalaycı were *c*Z and iP-types; saline conditions caused no significant changes in the total *t*Z, *c*Z and iP contents of the other two cultivars. A previous study found that total *t*Z and iP levels increased in tomato plants under salt stress^24^. However, CK levels reportedly fell in maize grown in media containing 2% NaCl^25^. Ribosides were the most salt-sensitive CK forms; their levels increased with the salt concentration in all cultivars. This increase was usually accompanied by a drop in the levels of endogenous O-glucosides and sometimes also 9-glucosides. Interestingly, levels of total free bases increased approximately twofold at 150 mM NaCl in Kalaycı and Erginel, while slightly decreasing in Akhisar. In keeping with these findings, drought-tolerant *Brassica oleracea* var. *acephala* (kale) exhibited significant accumulation of total CK bases (i.e. active CK forms) under salt stress^50^. These results suggest that the superior salt tolerance of Kalaycı is partly due to its accumulation of CKs under severe salt stress. Both SA treatments reduced the total CK content of Akhisar and Erginel, but increased that in Kalaycı. Combined SA and NaCl treatments generally increased CK levels, particularly at 300 mM NaCl. The cultivar-dependent effects of SA co-treatment under salt stress differed from those of SA pre-treatment. However, it appears that CK accumulation may enhance resistance to salt stress^51^. Additionally, SA pre-treatment was more effective at increasing CK levels in the more resistant variety (Kalaycı), whereas SA co-treatment was more effective at increasing CK levels in the moderately tolerant (Erginel) and sensitive (Akhisar) barley cultivars.

JA is a signaling molecule that is strongly associated with stress induced by trauma and pathogens, but it is also associated with abiotic stresses^52^. SA, ethylene, auxin, and other plant hormones interact with JA to regulate plant adaptation to the environment; however, JA and SA-mediated signaling pathways are mainly related to plant resistance to external damage and pathogen infection^53^. We therefore investigated changes in endogenous JA levels due to SA treatment under salt stress. Our results suggest that endogenous JA levels are sensitive to both exogenous SA and salinity, and may differ between genotypes and NaCl concentrations, respectively. Anderson et al.^54^ concluded that there is an antagonistic relationship between SA and JA levels, and Singh and Gautam^55^ also reported that SA can act as a negative allosteric effector of JA. We observed no such direct antagonist relationship between SA and JA in barley. In contrast to the results presented here, JA levels decreased in salt-sensitive tomatoes exposed to NaCl for 24 h^56^.

Ethylene is a gaseous hormone that is considered to be a stress hormone similar to ABA. The effects of salt stress on ethylene production in the studied barley cultivars were relatively clear-cut: ethylene levels increased with salinity, sometimes quite sharply (as in the case of the Akhisar cultivar at 300 mM NaCl). SA treatment also induced small to significant increases in ethylene production in the barley cultivars, but SA treatment at 300 mM NaCl (pSA300 and cSA300) generally reduced ethylene levels. Some previous studies have measured endogenous ethylene under salt stress. For example, the accumulation of ethylene in an ozone-tolerant *Populus* cultivar was found to be much lower than in a sensitive one^57^. Additionally, SA was reported to inhibit ethylene biosynthesis in mung bean^58^ and rice leaves^59^, and an antagonistic relationship between ABA and ethylene was identified in Arabidopsis seeds^60^. However, no such antagonistic relationship was apparent in our results.

## Conclusion

This study investigated the effect of treatment with exogenous salicylic acid (SA) in three barley cultivars (*Hordeum vulgare* L. cv. Akhisar (sensitive), cv. Erginel (moderate) and cv. Kalaycı (tolerant)) with different salinity tolerances under salt stress conditions. Changes in the cultivars’ relative leaf water content (RWC), growth parameters, proline content, chlorophyll *a/b* (Chl *a/b*) ratio, and lipid peroxidation (TBARS) were monitored, along with the levels of selected endogenous phytohormones and the activity of enzymes involved in detoxifying reactive oxygen species (ROS) such as superoxide dismutase (SOD), peroxidase (POX), catalase (CAT), ascorbate peroxidase (APX), and glutathione reductase (GR). This comparative analysis of osmoregulation, biomass, chlorophyll content and lipid peroxidation revealed that the Kalaycı cultivar adapted better to high salinity. Exogenous SA treatment increased antioxidant defense enzyme activity, enabling ROS detoxification. However, the effects of SA treatment differed between the barley genotypes and depended on the treatment timing. Notably, some SA treatment regimes helped maintain plant growth and alleviated the adverse impact of oxidative stress while also changing endogenous phytohormone levels and the way they responded to salt stress. Pre-treatment with SA before exposure to saline conditions mitigated the negative effects of salt stress and facilitated redox regulation and antioxidant defense in Kalaycı, whereas SA treatment applied at the same time as the salt stress conferred better protection in Erginel. Finally, this work demonstrates that the reinforcement of antioxidant defense systems in plants is closely tied to changes in endogenous phytohormone levels, which play key roles in adaptation to salt stress.

## Material and methods

### Plant material and stress applications

Seeds of three barley (*Hordeum vulgare* L.; Poaceae; 2n = 14) cultivars were obtained from the Transitional Zone Agricultural Research Institute, Eskişehir, Turkey (Kalaycı-97 and Erginel-90) and the Aegean Agricultural Research Institute, İzmir, Turkey (Akhisar-98). The seeds were surface sterilized with 70% ethanol for 5 min, rinsed in sterile deionised water, and then immersed in 5% commercial bleach for 15 min. To remove residual bleach, the seeds were washed at least five times with sterile deionised water. Afterwards, they were germinated in darkness at 22 °C and 70% humidity for two days. After germination, the barley seedlings were grown at 22/18 °C (day/night) under 300 μmol m^−2^ s^−1^ irradiance for a 16 /8 h (day/night) period at 70% humidity in perlite-filled pots irrigated every other day with half-strength Hoagland’s solution^61^. After 16 days, the seedlings were randomly divided into nine groups: C (untreated control plants grown under non-saline conditions); 150 (growth in 150 mM NaCl for 4 days); 300 (growth in 300 mM NaCl for 4 days); pSAC (pre-treatment with 0.5 mM SA for 24 h followed by growth under non-saline conditions); pSA150 (pre-treatment with 0.5 mM SA followed by growth in 150 mM NaCl); pSA300 (pre-treatment with 0.5 mM SA for 24 h followed by growth in 300 mM NaCl); cSAC (continuous treatment with 0.5 mM SA for 4 days while growing under non-saline conditions); cSA150 (continuous treatment with 0.5 mM SA for 4 days while growing in 150 mM NaCl) and cSA300 (continuous treatment with 0.5 mM SA for 4 days while growing in 300 mM NaCl). SA and/or NaCl were added to the Hoagland nutrient solution as appropriate. An SA concentration of 0.5 mM was chosen on the basis of preliminary experiments that measured the ability of various SA concentrations (0.1, 0.5 and 1.0 mM) to induce radicle growth and rescue seedlings from the detrimental effects of NaCl. Root and shoot samples were harvested separately on day 21, then immediately frozen in liquid nitrogen (− 196 °C) and stored in a freezer at − 80 °C until analysis.

### Growth measurements

Seven seedlings were randomly selected from each experimental group and divided into shoots and roots. Fresh weight (FW) and shoot length (SL) were measured immediately after harvesting. Shoot length was determined by measuring the average length of the longest leaves. The shoots were then dried at 80 °C for 48 h to determine their dry weight (DW).

### Relative leaf water content

Leaves from each experimental group were weighed to determine their fresh weight (FW). They were then placed in deionized water at 4 °C for 16 h, after which the turgid tissue was briefly dried between two filter papers to remove excess water and the leaf samples were reweighed to determine the weight of the fully turgid sheets (TW). The dry weight (DW) was measured after drying the leaves in an oven at 70 °C for 48 h. The relative water content (RWC) of the leaves was calculated using the following expression:

$$ {\text{RWC }}\left( \% \right) \, = \, \left[ {\left( {{\text{FW }}{-}{\text{ DW}}} \right) \, / \, \left( {{\text{TW}} - {\text{DW}}} \right)} \right] \, \times { 1}00 $$

### Chlorophyll content

Chlorophylls (Chl) were extracted in 80% acetone for 48 h in the dark. The absorbance of the Chl extracts at 663 and 645 nm was then measured using a UV–VIS spectrophotometer (Thermo, Evolution 100, UK) and the Chl *a/b* ratio was calculated using the following expressions^62^:

$$ {\text{Chl}}\;a = \, \Delta {\text{A}}_{{{663}}} \times { 12}.{7 }{-} \, \Delta {\text{A}}_{{{645}}} \times { 2}.{69} \;\;\;\;\; {\text{Chl}}\;b = \, \Delta {\text{A}}_{{{645}}} \times { 22}.{9 }{-} \, \Delta {\text{A}}_{{{663}}} \times { 4}.{68} $$

### Determination of proline content

The free proline content was determined according to Bates et al.^63^. Leaf samples were homogenized in 3% sulfosalicylic acid and the homogenate was filtered through Whatman’s No. 2 filter paper. The filtered extracts were then tested for proline content using the acid-ninhydrin method. Proline contents were determined with a UV–VIS spectrophotometer at 520 nm (Thermo, Evolution 100, UK) using a standard curve generated by analysing proline solutions of known concentration (μmol proline g^-1^ FW).

### Estimation of lipid peroxidation

Levels of thiobarbituric acid-reactive substances (TBARS) were measured to determine the concentration of the lipid peroxidation end product malondialdehyde, as described by Heath and Packer^64^. The TBARS content was calculated by spectrophotometric measurement at 532 nm, correcting for nonspecific turbidity by subtracting the absorbance at 600 nm. The TBARS concentration was calculated using an extinction coefficient of 155 mM^−1^ cm^−1^.

### Antioxidant enzyme extractions and activity assays

To determine antioxidant enzyme activities, leaf samples were ground to fine powder in liquid nitrogen with an ice-cold mortar and pestle and then homogenized in ice-cold 50 mM potassium phosphate buffer (pH 7.0) containing 1 mM EDTA and 1% polyvinylpyrrolidone (PVP). Ascorbate (2 mM) was added to the ascorbate peroxidase (APX) homogenization buffer. All enzyme extraction steps were performed at 4 °C. The homogenized samples were centrifuged at 15,000×*g* for 15 min and the resulting supernatants were used to determine protein contents and enzyme activities. Protein content was determined using bovine serum albumin (BSA) as a standard^65^. Activities were determined for five enzymes: superoxide dismutase (SOD, EC.1.15.1.1), peroxidase (POX, EC.1.11.1.7), catalase (CAT, EC 1.11.1.6), ascorbate peroxidase (APX, EC 1.11.1.11), and glutathione reductase (GR, EC 1.6.4.2).

SOD activity was determined as described by Beauchamp and Fridovich^66^, using reaction mixtures containing 50 mM potassium phosphate buffer (pH 7.0), 0.1 mM EDTA, 13 mM methionine, 0.075 mM NBT, 0.002 mM riboflavin, and 50 μL enzyme extract. This assay involves measuring the inhibition of the photochemical reduction of nitro blue tetrazolium (NBT) at 560 nm; one unit of SOD was defined as the amount of enzyme that inhibits NBT photoreduction by 50%. The reaction was initiated by adding riboflavin and irradiating the mixture at 300 μmol m^−2^ s^−1^ for 10 min. POX activity was assayed as described by Mika and Lüthje^67^, using reaction mixtures containing 25 mM sodium acetate buffer (pH 5.0), 10 mM guaiacol, and 10 mM H_2_O_2_. This assay involves monitoring the reaction mixture’s absorbance at 470 nm over 1 min. POX activity was calculated using an absorbance coefficient of 26.6 mM^−1^ cm^−1^; one unit of POX activity was defined as 1 μmol decomposed H_2_O_2._min^−1^. CAT activity was determined as described by Aebi^68^, using reaction mixtures containing 50 mM potassium phosphate buffer (pH 7.0) and 10 mM H_2_O_2_. The mixtures’ absorbance at 240 nm was monitored over 3 min; one unit of CAT activity was defined as 1 µmol H_2_O_2_ decomposed min^−1^, and activity was calculated using an extinction coefficient of 39.4 mM^−1^ cm^−1^. APX activity was measured as described by Nakano and Asada^69^, using reaction mixtures containing 50 mM sodium phosphate buffer (pH 7.0), 250 µM ascorbate, and 5 mM H_2_O_2_. This assay involves measuring the decrease in absorbance at 290 nm as ascorbate is oxidized. The concentration of oxidized ascorbate was calculated using an extinction coefficient of 2.8 mM^−1^ cm^−1^ and one unit of APX activity was defined as 1 µmol ascorbate oxidized in 1 min. GR activity was determined as described by Foyer and Halliwell^70^, using reaction mixtures containing 50 mM Tris–HCl buffer (pH 7.6), 5 mM NADPH, and 10 mM glutathione disulfide (GSSG, oxidized glutathione). The assay depends on the decrease in absorbance at 340 nm, and the absorbance coefficient is taken to be 6.2 mM^−1^ cm^−1^. One unit of GR was defined as 1 µmol GSSG reduced in 1 min.

## Phytohormone analysis

Concentrations of endogenous phytohormones including indole-3-acetic acid (IAA), *trans*-zeatin (*t*Z), isopentenyladenine (iP), abscisic acid (ABA), jasmonic acid (JA), and ethylene were determined in the leaves of three barley cultivars with differing salinity tolerance. All measurements were repeated three times. Endogenous IAA was purified from leaves and roots and quantified as described by Pencik et al.^71^. Separation was performed using an ultra-performance liquid chromatograph (Acquity UPLC; Waters) equipped with a Symmetry C18 column (5 µm, 2.1 mm × 150 mm; Waters), and the effluent was introduced into the electrospray ion source of a Quatro micro API tandem quadrupole mass spectrometer (Waters). Extraction and purification of endogenous CKs were performed according to Novák et al.^72^, and their levels were determined by ultra-high performance liquid chromatography–electrospray tandem mass spectrometry (UHPLC–MS/MS)^73^. Endogenous ABA was purified by solid-phase extraction on Oasis HLB cartridges (60 mg, 3 mL; Waters) with quantification by a UHPLC–MS/MS system^74^. Endogenous JA was determined after overnight extraction with 80% (v/v) methanol using a UHPLC–MS/MS method^75^. All the analytes were measured in MRM mode using optimized cone voltages and collision energies for diagnosis of each phytohormone. Stable isotope-labelled internal standards were used as references to quantify endogenous phytohormone levels. Data analysis was performed using MassLynx™ 4.1 (Waters, Manchester, UK) and the phytohormones were quantified by the standard isotope-dilution method using three technical replicates per biological sample. Endogenous ethylene concentrations were determined using the gas chromatography-flame ionization detector (GC-FID) method^76^. Plants in each of the nine groups were placed separately in gas-tight plastic bags containing 10 ml growth medium as described in the experimental design. The bags were then tightly sealed with a rubber septum and placed in light for 1 h. Subsequently, 1 ml of air was taken from each bag and analyzed using a Finnigan Trace GC Ultra equipped with a FID detector and a 50 m capillary column (HP-AL/S stationary phase, 15 μm, i.d. = 0.535).

### Statistical analysis

All analyses were performed according to a completely randomized design. Two biological replicates with three technical replicates each (n = 6) were analyzed for each experimental condition. The results obtained are expressed as means and error bars are used to show the standard error of the mean (± SEM) and originate from 2-independent experiments. All data obtained were subjected to one-way analysis of variance (ANOVA) and significant differences between treatments were compared using Duncan’s Multiple Range Test (DMRT). A significance threshold of *P* < 0.05 was applied.

## Supplementary information

Supplementary file1

## Data Availability

Data used in this manuscript will be available to the public.
