# Supplementary material for: Timing-dependent effects of salicylic acid treatment on phytohormonal changes, ROS regulation, and antioxidant defense in salinized barley (Hordeum vulgare L.)
Source: Sci Rep. 2020 Aug 17;10:13886. doi: 10.1038/s41598-020-70807-3 (PMC7431421; doi:10.1038/s41598-020-70807-3)
Supplement: Supplementary file 1 — Supplementary file1 [file 41598_2020_70807_MOESM1_ESM.pdf]

Timing-Dependent Effects of Salicylic Acid Treatment on Phytohormonal Changes, ROS Regulation, and Antioxidant Defense in Salinized Barley (*Hordeum vulgare* L.)

Hülya Torun[1,2*], Ondřej Novák[3], Jaromír Mikulík[3], Aleš Pěnčík[3], Miroslav Strnad[3], Faik Ahmet Ayaz[2]

[1]Faculty of Agriculture and Natural Science, Düzce University, Düzce, Turkey
[2]Faculty of Science, Karadeniz Technical University, Trabzon, Turkey
[3]Laboratory of Growth Regulators, Faculty of Science, Palacký University and Institute of Experimental Botany, The Czech Academy of Sciences, Šlechtitelů 27, CZ-78371 Olomouc, Czech Republic

[*]Correspondence
Hülya Torun
hulyatorun@duzce.edu.tr

Supplementary Table S1

Effects of pre- and co-treatment with salicylic acid (SA) on growth parameters (shoot length (L; cm), shoot fresh weight (FW; g) and dry weight (DW; g)) of *Hordeum vulgare* L. cultivars grown under control (C) and saline (150 and 300 mM NaCl) conditions. Values are means ± SE (n = 7). Asterisks denote significant differences from controls ($P < 0.05$).

	**C**	**150**	**300**	**pSAC**	**pSA150**	**pSA300**	**cSAC**	**cSA150**	**cSA300**
	**Akhisar**								
**L**	48.0 ± 0.5	35.7 ± 5.1*	35.5 ± 1.6*	47.0 ± 4.7	38.8 ± 3.3*	40.8 ± 4.4*	47.7 ± 1.1	41.7 ± 2.3*	41.3 ± 1.2*
**FW**	2.33 ± 0.22	1.17 ± 0.41*	0.93 ± 0.24*	1.73 ± 0.50*	1.48 ± 0.23*	1.32 ± 0.25*	1.93 ± 0.18	1.40 ± 0.25*	1.40 ± 0.01*
**DW**	0.27 ± 0.06	0.15 ± 0.05*	0.14 ± 0.04*	0.17 ± 0.04*	0.23 ± 0.01	0.19 ± 0.04*	0.20 ± 0.05	0.31 ± 0.10	0.33 ± 0.03
	**Erginel**								
**L**	43.8 ± 1.52	37.9 ± 1.01*	34.9 ± 3.96*	45.3 ± 1.88	41.4 ± 1.91	37.8 ± 4.68*	47.6 ± 1.56*	40.3 ± 3.25	32.3 ± 0.07*
**FW**	3.00 ± 0.11	1.26 ± 0.16*	1.41 ± 0.17*	3.67 ± 0.34*	2.13 ± 0.08*	1.77 ± 0.63*	2.90 ± 0.83	2.15 ± 0.52	1.80 ± 0.26
**DW**	0.36 ± 0.01	0.17 ± 0.02*	0.17 ± 0.05*	0.45 ± 0.05*	0.29 ± 0.01*	0.34 ± 0.13	0.52 ± 0.21*	0.36 ± 0.12	0.44 ± 0.02
	**Kalaycı**								
**L**	44.8 ± 1.84	38.5 ± 1.15*	43.0 ± 0.65	44.2 ± 0.74	44.8 ± 3.75	41.6 ± 4.10*	45.3 ± 1.25	45.5 ± 0.21	42.7 ± 1.62*
**FW**	3.33 ± 0.22	2.13 ± 0.59*	1.93 ± 0.16*	2.15 ± 0.74*	2.18 ± 0.41*	2.09 ± 0.22*	2.64 ± 0.74*	2.06 ± 0.09*	2.14 ± 0.27*
**DW**	0.52 ± 0.04	0.39 ± 0.14*	0.31 ± 0.04*	0.19 ± 0.02*	0.40 ± 0.13*	0.29 ± 0.06*	0.29 ± 0.08*	0.25 ± 0.03*	0.42 ± 0.07*

Supplementary Table S2

Effect of pre- and co-treatment with salicylic acid (SA) on endogenous cytokinin (CK) levels of *Hordeum vulgare* L. cultivars under control (C) and saline (150 mM and 300 mM NaCl) conditions. Values are means ± SE (*n* = 7). Asterisks denote significant differences from controls ($P < 0.05$).

Cytokinin type	Endogenous cytokinin content (pmol g^{-1} FW)								
	**C**	**150**	**300**	**pSAC**	**pSA150**	**pSA300**	**cSAC**	**cSA150**	**cSA300**
	**Akhisar**								
Total CK Bases	3.57 ± 0.22	3.02 ± 0.25*	2.34 ± 0.81*	4.09 ± 0.53	2.41 ± 0.15	1.93 ± 0.41	3.50 ± 0.56	1.96 ± 0.02	1.65 ± 0.41
Total CK Ribosides	3.28 ± 0.41	5.40 ± 0.16*	7.97 ± 2.05*	6.89 ± 1.81*	3.52 ± 0.07	5.03 ± 0.63	8.60 ± 1.81*	3.01 ± 0.37	3.42 ± 0.64
Total CK Nucleotides	2.55 ± 0.54	0.65 ± 0.00*	1.75 ± 0.20*	2.03 ± 0.26*	0.99 ± 0.01	1.43 ± 0.10	1.02 ± 0.07*	1.03 ± 0.05	2.27 ± 0.34
Total CK *O*-glucosides	50.68 ± 1.82	45.18 ± 3.90	39.77 ± 1.27*	38.32 ± 0.80*	42.19 ± 1.90	57.57 ± 6.87	49.55 ± 3.73	49.44 ± 1.69	61.25 ± 4.70
Total CK 9-glucosides	6.75 ± 0.14	3.05 ± 0.21*	2.54 ± 0.07*	5.12 ± 0.15*	3.64 ± 0.11	2.98 ± 0.09	2.26 ± 0.08	3.66 ± 0.08	2.93 ± 0.18
Total CK	66.83 ± 1.77	57.29 ± 4.02*	54.37 ± 4.40*	56.44 ± 1.95*	52.75 ± 1.72*	68.94 ± 5.85	64.93 ± 1.38	59.10 ± 1.95*	71.52 ± 4.30
	**Erginel**								
Total CK Bases	8.99 ± 1.87	22.14 ± 0.16*	9.15 ± 0.47	7.51 ± 0.15*	7.63 ± 0.47	12.71 ± 0.70	7.86 ± 0.36	17.02 ± 1.23	12.04 ± 0.21
Total CK Ribosides	31.68 ± 6.46	68.75 ± 2.53*	33.12 ± 3.16	36.76 ± 2.48	31.27 ± 1.09	57.26 ± 0.88	35.55 ± 1.72	95.49 ± 5.85	50.35 ± 3.09
Total CK Nucleotides	2.96 ± 0.06	1.34 ± 0.02*	2.34 ± 0.16*	2.06 ± 0.56*	1.37 ± 0.08	2.13 ± 0.41	2.08 ± 0.14	3.19 ± 0.21	1.85 ± 0.45
Total CK *O*-glucosides	65.49 ± 2.16	39.04 ± 0.86*	53.04 ± 1.33*	51.19 ± 1.53*	44.96 ± 0.04	56.19 ± 4.43	44.39 ± 1.51	55.49 ± 1.40	61.31 ± 1.38
Total CK 9-glucosides	6.90 ± 0.10	6.95 ± 0.14	3.41 ± 0.07*	4.57 ± 0.06*	5.44 ± 0.09	6.04 ± 0.12	7.16 ± 0.42	4.81 ± 0.04	4.89 ± 0.32
Total CK	116.0 ± 6.1	138.2 ± 1.7*	101.1 ± 5.1*	102.1 ± 3.7*	90.7 ± 1.5*	134.3 ± 4.5*	97.0 ± 0.3*	176.0 ± 8.7*	130.4 ± 4.6*
	**Kalaycı**								
Total CK Bases	2.36 ± 0.00	5.21 ± 1.64*	2.85 ± 0.02*	2.81 ± 0.00*	5.77 ± 0.83	9.80 ± 2.07	5.99 ± 0.26	4.12 ± 0.62	4.42 ± 0.38
Total CK Ribosides	1.74 ± 0.24	13.58 ± 4.28*	14.06 ± 1.62*	9.40 ± 1.30*	21.45 ± 4.11	28.04 ± 7.87	16.66 ± 1.21	7.56 ± 1.10	10.48 ± 1.76
Total CK Nucleotides	0.70 ± 0.05	2.13 ± 0.18*	3.58 ± 0.58*	2.66 ± 0.20*	2.51 ± 0.15	7.64 ± 0.40	2.61 ± 0.44	0.95 ± 0.06	1.63 ± 0.31
Total CK *O*-glucosides	44.99 ± 1.93	46.72 ± 1.54	64.37 ± 4.58*	59.08 ± 5.18*	45.69 ± 0.26	75.62 ± 0.17	57.43 ± 4.03	48.12 ± 0.95	65.23 ± 0.09
Total CK 9-glucosides	6.80 ± 0.06	9.16 ± 0.11*	7.51 ± 0.17*	4.99 ± 0.03*	6.17 ± 0.17	6.45 ± 0.16	4.48 ± 0.04	5.52 ± 0.15	4.47 ± 0.02
Total CK	56.60 ± 2.29	76.79 ± 4.67*	92.38 ± 6.97*	78.94 ± 6.72*	81.59 ± 4.99*	127.5 ± 9.5*	87.18 ± 5.98*	66.27 ± 0.67*	86.24 ± 2.52*

Supplementary Table S3

Effects of pre- and co-treatment with salicylic acid (SA) on endogenous cytokinin levels of *Hordeum vulgare* L. 'Akhisar' leaves grown under control (C) and saline (150 and 300 mM NaCl) conditions. Values are means ± SE (*n* = 7).

**Cytokinin type**	**Endogenous cytokinin content (pmol g^{-1} FW)**								
	**C**	**150**	**300**	**pSAC**	**pSA150**	**pSA300**	**cSAC**	**cSA150**	**cSA300**
***t*Z**	2.61 ± 0.04	1.28 ± 0.25	0.81 ± 0.00	1.80 ± 0.09	1.32 ± 0.01	0.71 ± 0.00	0.87 ± 0.18	1.12 ± 0.11	0.82 ± 0.00
***t*ZR**	0.44 ± 0.00	0.14 ± 0.01	0.11 ± 0.02	0.39 ± 0.05	0.22 ± 0.02	0.09 ± 0.00	0.06 ± 0.01	0.21 ± 0.02	0.05 ± 0.00
***t*Z9G**	6.37 ± 0.11	2.51 ± 0.04	2.10 ± 0.00	4.54 ± 0.09	2.98 ± 0.08	2.37 ± 0.08	1.87 ± 0.05	3.08 ± 0.16	2.47 ± 0.21
***t*ZOG**	0.99 ± 0.04	0.30 ± 0.08	0.18 ± 0.04	0.34 ± 0.01	0.18 ± 0.00	0.20 ± 0.05	0.15 ± 0.01	0.34 ± 0.02	0.18 ± 0.02
***t*ZROG**	0.53 ± 0.01	0.11 ± 0.04	<LOD	0.98 ± 0.20	0.29 ± 0.09	0.28 ± 0.05	<LOD	0.40 ± 0.02	0.39 ± 0.05
***t*ZR'5MP**	0.43 ± 0.08	0.11 ± 0.00	0.16 ± 0.03	0.69 ± 0.12	0.24 ± 0.01	<LOD	0.21 ± 0.00	0.45 ± 0.01	0.23 ± 0.01
**Total *t*Z**	**11.37 ± 0.05**	**4.40 ± 0.24**	**2.95 ± 0.31**	**8.74 ± 0.37**	**5.23 ± 0.02**	**3.29 ± 0.43**	**3.06 ± 0.02**	**5.60 ± 0.24**	**3.73 ± 0.68**
***c*Z**	0.76 ± 0.17	1.52 ± 0.03	1.81 ± 0.38	1.96 ± 0.61	0.91 ± 0.15	1.42 ± 0.08	2.47 ± 0.34	0.63 ± 0.12	1.07 ± 0.01
***c*ZR**	1.11 ± 0.23	3.37 ± 0.14	5.52 ± 1.47	4.22 ± 1.46	1.51 ± 0.03	3.11 ± 0.04	5.81 ± 1.48	1.72 ± 0.44	2.59 ± 0.43
***c*Z9G**	0.08 ± 0.00	0.05 ± 0.00	0.05 ± 0.00	0.08 ± 0.01	0.06 ± 0.00	0.07 ± 0.01	0.04 ± 0.00	0.06 ± 0.01	0.06 ± 0.00
***c*ZOG**	46.36 ± 1.49	39.51 ± 3.67	31.82 ± 0.89	33.64 ± 0.89	38.15 ± 1.56	48.42 ± 6.32	45.27 ± 3.91	44.74 ± 1.69	49.20 ± 3.93
***c*ZROG**	2.80 ± 0.28	5.25 ± 0.20	7.77 ± 0.42	3.36 ± 0.11	3.57 ± 0.24	8.67 ± 0.56	4.12 ± 0.19	3.96 ± 0.00	11.48 ± 0.71
***c*ZR'5MP**	1.96 ± 0.65	0.51 ± 0.04	1.48 ± 0.19	1.14 ± 0.15	0.65 ± 0.03	1.32 ± 0.10	0.89 ± 0.16	0.47 ± 0.05	1.89 ± 0.32
**Total *c*Z**	**53.07 ± 1.53**	**50.22 ± 3.73**	**48.44 ± 3.34**	**44.39 ± 1.22**	**44.85 ± 1.70**	**63.02 ± 6.85**	**58.60 ± 1.74**	**51.59 ± 2.21**	**66.28 ± 3.88**
**iP**	0.20 ± 0.01	0.22 ± 0.04	0.13 ± 0.03	0.34 ± 0.02	0.18 ± 0.01	0.16 ± 0.02	0.16 ± 0.04	0.20 ± 0.01	0.17 ± 0.00
**iPR**	1.74 ± 0.18	1.89 ± 0.31	2.34 ± 0.60	2.28 ± 0.30	1.78 ± 0.07	1.83 ± 0.59	2.73 ± 0.32	1.08 ± 0.09	0.78 ± 0.21
**iP9G**	0.29 ± 0.03	0.49 ± 0.16	0.39 ± 0.08	0.50 ± 0.05	0.61 ± 0.03	0.54 ± 0.00	0.35 ± 0.03	0.52 ± 0.07	0.40 ± 0.03
**iPR'5MP**	0.16 ± 0.02	0.08 ± 0.02	0.12 ± 0.04	0.20± 0.02	0.11 ± 0.03	0.11 ± 0.00	0.03 ± 0.01	0.10 ± 0.02	0.15 ± 0.03
**Total iP**	**2.39± 0.19**	**2.67 ± 0.53**	**2.98 ± 0.74**	**3.31 ± 0.35**	**2.68 ± 0.00**	**2.63 ± 0.57**	**3.27 ± 0.34**	**1.91 ± 0.02**	**1.50 ± 0.26**

Below the limit of detection (< LOD), *trans*-Zeatin (*t*Z), *trans*-Zeatin riboside (*t*ZR), *trans*-Zeatin-9-glucoside (*t*Z9G), *trans*-Zeatin-*O*-glucoside (*t*ZOG), *trans*-zeatin-*O*-glucoside riboside (*t*ZROG), *trans*-Zeatin-9-riboside-5′-monophosphate (*t*ZR5′MP), *cis*-Zeatin (*c*Z), *cis*-zeatin riboside (*c*ZR), *cis*-Zeatin-9-glucoside (*c*Z9G), cis-Zeatin-*O*-glucoside (*c*ZOG), *cis*-Zeatin-*O*-glucoside riboside (*c*ZROG), *cis*-Zeatin-9-riboside-5′-monophosphate (cZR5′MP), N^6-isopentenyladenine (iP), N^6-isopentenyladenosine (iPR), N^6-isopentenyladenine-9-glucose (iP9G) and N^6-isopentenyladenosine-5′-monophosphate (iPR5′MP).

Supplementary Table S4

Effects of pre- and co-treatment with salicylic acid (SA) on endogenous cytokinin levels of *Hordeum vulgare* L. 'Erginel' leaves grown under control (C) and saline (150 and 300 mM NaCl) conditions. Values are means ± SE (*n* = 7).

**Cytokinin type**	**Endogenous cytokinin content (pmol g^{-1} FW)**								
	**C**	**150**	**300**	**pSAC**	**pSA150**	**pSA300**	**cSAC**	**cSA150**	**cSA300**
***t*Z**	0.80 ± 0.01	1.53 ± 0.17	0.87 ± 0.01	0.65 ± 0.05	0.77 ± 0.01	0.95 ± 0.00	0.10 ± 0.08	1.46 ± 0.25	<LOD
***t*ZR**	0.20 ± 0.02	0.29 ± 0.00	0.22 ± 0.00	0.11 ± 0.00	0.15 ± 0.02	0.17 ± 0.02	0.16 ± 0.03	0.21 ± 0.00	0.24 ± 0.02
***t*Z9G**	6.60 ± 0.11	6.69 ± 0.10	3.10 ± 0.11	4.28 ± 0.02	5.05 ± 0.17	5.85 ± 0.16	6.64 ± 0.35	4.07 ± 0.11	4.52 ± 0.35
***t*ZOG**	0.92 ± 0.15	0.41 ± 0.04	0.23 ± 0.00	0.35 ± 0.00	0.50 ± 0.02	0.41 ± 0.12	0.64 ± 0.06	0.43 ± 0.01	0.38 ± 0.05
***t*ZROG**	1.05 ± 0.05	0.80 ± 0.10	<LOD	<LOD	0.37 ± 0.07	0.32 ± 0.01	0.31 ± 0.04	0.95 ± 0.27	0.40 ± 0.01
***t*ZR'5MP**	0.38 ± 0.03	<LOD	0.15 ± 0.00	0.14 ± 0.00	0.19 ± 0.04	0.21 ± 0.07	0.18 ± 0.00	0.53 ± 0.08	0.32 ± 0.06
**Total *t*Z**	**9.95 ± 0.14**	**9.72 ± 0.40**	**4.50 ± 0.02**	**5.45 ± 0.10**	**7.05 ± 0.10**	**7.43 ± 0.50**	**8.93 ± 0.22**	**7.66 ± 0.33**	**5.86 ± 0.35**
***c*Z**	7.85 ± 1.79	20.19 ± 0.31	8.07 ± 0.45	6.60 ± 0.19	6.57 ± 0.44	11.93 ± 0.25	6.52 ± 0.41	14.83 ± 0.95	11.72 ± 0.21
***c*ZR**	23.42 ± 4.65	46.19 ± 2.04	25.49 ± 2.02	24.15 ± 0.72	23.60 ± 0.62	39.20 ± 0.40	25.55 ± 1.15	55.02 ± 0.97	33.82 ± 2.13
***c*Z9G**	0.06 ± 0.00	0.04 ± 0.00	0.03 ± 0.01	0.04 ± 0.01	0.05 ± 0.01	0.06 ± 0.01	0.04 ± 0.00	0.07 ± 0.01	0.07 ± 0.00
***c*ZOG**	56.09 ± 1.70	30.86 ± 0.86	43.90 ± 0.89	42.86 ± 1.29	37.16 ± 0.09	45.74 ± 4.24	36.86 ± 1.16	42.22 ± 1.10	48.49 ± 1.82
***c*ZROG**	7.42 ± 0.36	6.97 ± 0.14	8.92 ± 0.44	7.98 ± 0.24	6.92 ± 0.04	9.73 ± 0.04	6.58 ± 0.44	11.89 ± 0.03	12.05 ± 0.48
***c*ZR'5MP**	2.43 ± 0.01	1.18 ± 0.04	2.05 ± 0.06	1.88 ± 0.46	1.06 ± 0.10	1.86 ± 0.28	1.92 ± 0.03	2.47 ± 0.29	1.35 ± 0.33
**Total *c*Z**	**97.28 ± 4.37**	**105.42 ± 1.58**	**88.45 ± 3.87**	**83.53 ± 1.97**	**75.36 ± 1.02**	**108.50 ± 4.40**	**77.47 ± 0.01**	**126.49 ± 3.33**	**107.50 ± 3.35**
**iP**	0.33 ± 0.08	0.42 ± 0.01	0.21 ± 0.01	0.26 ± 0.01	0.29 ± 0.02	0.31 ± 0.02	0.25 ± 0.03	0.74 ± 0.03	0.32 ± 0.01
**iPR**	8.05 ± 1.79	22.27 ± 0.49	7.41 ± 1.13	12.49 ± 1.76	7.53 ± 0.45	17.90 ± 0.47	9.84 ± 0.54	40.25 ± 4.88	16.28 ± 0.94
**iP9G**	0.24 ± 0.00	0.23 ± 0.04	0.27 ± 0.03	0.25 ± 0.05	0.34 ± 0.09	0.13 ± 0.04	0.48 ± 0.07	0.68 ± 0.16	0.30 ± 0.03
**iPR'5MP**	0.15 ± 0.03	0.16 ± 0.02	0.22 ± 0.03	0.11 ± 0.03	0.12 ± 0.03	0.13 ± 0.00	0.08 ± 0.02	0.19 ± 0.00	0.18 ± 0.06
**Total iP**	**8.78 ± 1.90**	**23.08 ± 0.49**	**8.11 ± 1.20**	**13.12 ± 1.80**	**8.27 ± 0.59**	**18.40 ± 0.37**	**10.64 ± 0.52**	**41.86 ± 5.06**	**17.08 ± 0.86**

Below the limit of detection (< LOD), *trans*-Zeatin (*t*Z), *trans*-Zeatin riboside (*t*ZR), *trans*-Zeatin-9-glucoside (*t*Z9G), *trans*-Zeatin-*O*-glucoside (*t*ZOG), *trans*-zeatin-*O*-glucoside riboside (*t*ZROG), *trans*-Zeatin-9-riboside-5′-monophosphate (*t*ZR5′MP), *cis*-Zeatin (*c*Z), *cis*-zeatin riboside (*c*ZR), *cis*-Zeatin-9-glucoside (*c*Z9G), cis-Zeatin-*O*-glucoside (*c*ZOG), *cis*-Zeatin-*O*-glucoside riboside (*c*ZROG), *cis*-Zeatin-9-riboside-5′-monophosphate (cZR5′MP), N^6-isopentenyladenine (iP), N^6-isopentenyladenosine (iPR), N^6-isopentenyladenine-9-glucose (iP9G) and N^6-isopentenyladenosine-5′-monophosphate (iPR5′MP).

Supplementary Table S5

Effects of pre- and co-treatment with salicylic acid (SA) on endogenous cytokinin levels of *Hordeum vulgare* L. 'Kalaycı' leaves grown under control (C) and saline (150 and 300 mM NaCl) conditions. Values are means ± SE (*n* = 7).

**Cytokinin type**	**Endogenous cytokinin content (pmol g^{-1} FW)**								
	**C**	**150**	**300**	**pSAC**	**pSA150**	**pSA300**	**cSAC**	**cSA150**	**cSA300**
***t*Z**	1.76 ± 0.09	1.77 ± 0.54	0.69 ± 0.00	0.55 ± 0.02	1.24 ± 0.16	0.88 ± 0.12	1.15 ± 0.17	1.10 ± 0.00	0.71 ± 0.00
***t*ZR**	0.25 ± 0.02	0.61 ± 0.08	0.22 ± 0.02	0.12 ± 0.03	0.32 ± 0.03	0.29 ± 0.00	0.32 ± 0.01	0.26 ± 0.06	0.15 ± 0.04
***t*Z9G**	6.36 ± 0.07	8.31 ± 0.12	6.98 ± 0.04	4.52 ± 0.03	5.67 ± 0.02	6.02 ± 0.04	3.93 ± 0.11	5.14 ± 0.04	4.13 ± 0.02
***t*ZOG**	0.66 ± 0.02	0.99 ± 0.01	0.63 ± 0.04	0.45 ± 0.09	0.50 ± 0.02	0.78 ± 0.06	0.68 ± 0.00	0.58 ± 0.04	0.38 ± 0.03
***t*ZROG**	0.26 ± 0.06	1.09 ± 0.15	0.69 ± 0.06	0.34 ± 0.08	0.26 ± 0.00	1.05 ± 0.26	0.52 ± 0.04	<LOD	<LOD
***t*ZR'5MP**	0.30 ± 0.20	0.73 ± 0.09	0.38 ± 0.00	0.20 ± 0.02	<LOD	0.78 ± 0.10	0.32 ± 0.00	0.74 ± 0.03	<LOD
**Total *t*Z**	**9.59 ± 0.08**	**13.50 ± 0.78**	**9.25 ± 0.20**	**6.19 ± 0.08**	**7.99 ± 0.020**	**9.80 ± 0.49**	**6.75 ± 0.05**	**7.83 ± 0.08**	**5.01 ± 0.37**
***c*Z**	0.46 ± 0.11	3.08 ± 0.99	2.30 ± 0.34	2.10 ± 0.02	4.35 ± 0.66	8.60 ± 2.17	4.59 ± 0.42	2.83 ± 0.62	3.88 ± 0.70
***c*ZR**	0.77 ± 0.25	8.40 ± 3.15	8.01 ± 2.01	5.71 ± 0.78	13.96 ± 2.71	18.56 ± 4.69	11.24 ± 1.10	5.31 ± 0.86	7.70 ± 1.07
***c*Z9G**	0.04 ± 0.00	0.06 ± 0.00	0.05 ± 0.00	0.03 ± 0.01	0.03 ± 0.00	0.08 ± 0.01	0.04 ± 0.01	0.02 ± 0.00	0.04 ± 0.00
***c*ZOG**	40.38 ± 2.09	39.85 ± 1.93	50.50 ± 3.13	51.86 ± 6.17	38.99 ± 0.71	65.40 ± 0.05	50.55 ± 3.90	42.65 ± 1.43	55.75 ± 0.64
***c*ZROG**	3.70 ± 0.08	4.79 ± 0.27	12.55 ± 1.35	6.43 ± 1.01	5.94 ± 0.46	8.38 ± 0.10	5.69 ± 0.17	4.89 ± 0.44	9.10 ± 0.52
***c*ZR'5MP**	0.30 ± 0.11	1.26 ± 0.28	2.91 ± 0.60	2.26 ± 0.22	2.28 ± 0.10	6.20 ± 0.44	2.31 ± 0.28	0.21 ± 0.03	1.57 ± 0.37
**Total *c*Z**	**45.65 ± 2.26**	**57.45 ± 2.76**	**76.31 ± 7.45**	**68.38 ± 6.14**	**65.55 ± 3.22**	**107.21 ± 6.57**	**74.42 ± 5.85**	**55.92 ± 0.52**	**87.03 ± 2.26**
**iP**	0.14 ± 0.02	0.36 ± 0.10	0.20 ± 0.02	0.16 ± 0.04	0.18 ± 0.01	0.32 ± 0.02	0.25 ± 0.01	0.18 ± 0.00	0.19 ± 0.03
**iPR**	0.72 ± 0.01	4.57 ± 1.05	5.83 ± 0.40	3.57 ± 0.56	7.17 ± 1.37	9.18 ± 3.17	5.10 ± 0.12	1.99 ± 0.18	2.63 ± 0.66
**iP9G**	0.40 ± 0.01	0.79 ± 0.01	0.48 ± 0.13	0.44 ± 0.07	0.47 ± 0.15	0.35 ± 0.12	0.51 ± 0.06	0.36 ± 0.10	0.31 ± 0.01
**iPR'5MP**	0.10 ± 0.03	0.13 ± 0.01	0.30 ± 0.03	0.19 ± 0.01	0.23 ± 0.04	0.67 ± 0.13	0.14 ± 0.01	<LOD	0.14 ± 0.00
**Total iP**	**1.35 ± 0.06**	**5.85 ± 1.13**	**6.81 ± 0.28**	**4.36 ± 0.67**	**8.05 ± 1.57**	**10.53 ± 3.45**	**6.00 ± 0.08**	**2.53 ± 0.08**	**3.20 ± 0.63**

Below the limit of detection (< LOD), *trans*-Zeatin (*t*Z), *trans*-Zeatin riboside (*t*ZR), *trans*-Zeatin-9-glucoside (*t*Z9G), *trans*-Zeatin-*O*-glucoside (*t*ZOG), *trans*-zeatin-*O*-glucoside riboside (*t*ZROG), *trans*-Zeatin-9-riboside-5′-monophosphate (*t*ZR5′MP), *cis*-Zeatin (*c*Z), *cis*-zeatin riboside (*c*ZR), *cis*-Zeatin-9-glucoside (*c*Z9G), cis-Zeatin-*O*-glucoside (*c*ZOG), *cis*-Zeatin-*O*-glucoside riboside (*c*ZROG), *cis*-Zeatin-9-riboside-5′-monophosphate (cZR5′MP), N^6-isopentenyladenine (iP), N^6-isopentenyladenosine (iPR), N^6-isopentenyladenine-9-glucose (iP9G) and N^6-isopentenyladenosine-5′-monophosphate (iPR5′MP).
